# Tuning the donor–acceptor interactions in phase-segregated block molecules†‡

**DOI:** 10.1039/d1mh01141c

**Published:** 2021-09-20

**Authors:** Brigitte A. G. Lamers, Martin H. C. van Son, Freek V. de Graaf, Bart W. L. van den Bersselaar, Bas F. M. de Waal, Kazuki Komatsu, Hiroshi Sato, Takuzo Aida, José Augusto Berrocal, Anja R. A. Palmans, Ghislaine Vantomme, Stefan C. J. Meskers, E. W. Meijer

**Affiliations:** Institute for Complex Molecular Systems and Laboratory of Macromolecular and Organic Chemistry, Eindhoven University of Technology P.O. Box 513 5600 MB Eindhoven The Netherlands e.w.meijer@tue.nl; Geochemistry Research Center, Graduate School of Science, The University of Tokyo, 7-3-1 Hongo, Bunkyo-ku Tokyo 113-0033 Japan; RIKEN Center for Emergent Matter Science Wako Saitama 351-0198 Japan; Japan Science and Technology Agency (JST), Precursory Research for Embryonic Science and Technology (PRESTO), 4-1-8 Honcho Kawaguchi Saitama 332-0012 Japan; Department of Chemistry and Biotechnology, School of Engineering, The University of Tokyo, Bunkyo-ku Tokyo 113-8656 Japan; Adolphe Merkle Institute, Polymer Chemistry and Materials, University of Fribourg, Chemin des Verdiers 4 1700 Fribourg Switzerland; Institute for Complex Molecular Systems and Molecular Materials and Nanosystems, Eindhoven University of Technology P.O. Box 513 5600 MB Eindhoven The Netherlands

## Abstract

The assembly of donor–acceptor molecules *via* charge transfer (CT) interactions gives rise to highly ordered nanomaterials with appealing electronic properties. Here, we present the synthesis and bulk co-assembly of pyrene (Pyr) and naphthalenediimide (NDI) functionalized oligodimethylsiloxanes (oDMS) of discrete length. We tune the donor–acceptor interactions by connecting the pyrene and NDI to the same oligomer, forming a heterotelechelic block molecule (NDI-oDMSPyr), and to two separate oligomers, giving Pyr and NDI homotelechelic block molecules (Pyr-oDMS and NDI-oDMS). Liquid crystalline materials are obtained for binary mixtures of Pyr-oDMS and NDI-oDMS, while crystallization of the CT dimers occurred for the heterotelechelic NDI-oDMS-Pyr block molecule. The synergy between crystallization and phase-segregation coupled with the discrete length of the oDMS units allows for perfect order and sharp interfaces between the insulating siloxane and CT layers composed of crystalline CT dimers. We were able to tune the lamellar domain spacing and donor–acceptor CT interactions by applying pressures up to 6 GPa on the material, making the system promising for soft-material nanotechnologies. These results demonstrate the importance of the molecular design to tune the CT interactions and stability of a CT material.

## Abstract 2

A robust, soft material held together by charge transfer interactions is developed. The nanostructure dimensions and material properties can be tuned by molecular design or pressure.

## Graphical abstract

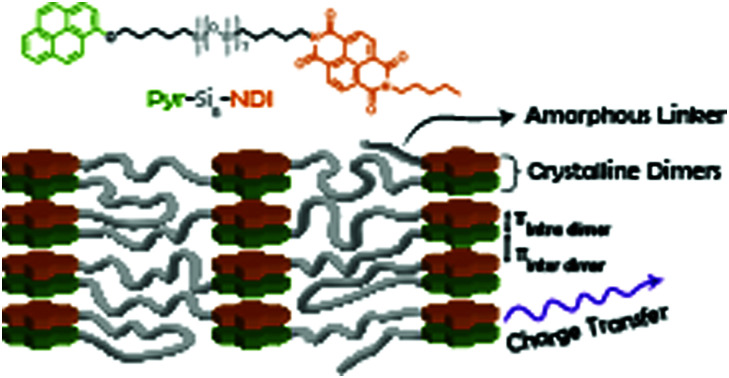

**New concepts**

Although donor–acceptor crystals are known for decades, the perfect arrangement is not achieved in soft (block copolymer) materials yet due to the disorder in polymeric materials. Here we introduce the concept of block molecules that are in-between crystals and polymers. They combine the perfect 2D crystallisation of lamellae while they still have the disordered part connecting the 2D crystalline parts. Pressure-induced tuning of the charge-transfer distance gives new directions for novel materials.

## Introduction

The alternate stacking of donor–acceptor (D–A) molecules driven by charge-transfer (CT) interactions has resulted in the development of various materials with fascinating electronic properties.^1–5^ The well-known examples are the many studies by Seth Marder and his group on the use of CT interactions in functional materials. His originality in bringing new concepts to materials science has inspired many of us.

A CT cocrystal that exhibits ferroelectricity at room temperature was reported by Stupp and Stoddart and coworkers.^6^ Alternatively, conductivity in CT cocrystals may be introduced upon applying pressure, inducing a phase-transition.^7^ Pressure-induced structural changes in these CT crystals result in a change of resistivity and thereby these systems become semiconducting, or even superconducting.^8–10^ For these electronic properties, the alternate stacking of donor and aceptor molecules is crucial and therefore the functional CT materials mostly comprise CT cocrystals.^11,12^

CT soft materials, such as gels,^13,14^ liquid crystals,^15,16^ and crosslinked supramolecular networks,^17,18^ have gained increasing attention recently.^19^ These materials are composed of hierarchical structures that are micelles, nanotubes or fibers, assembled *via* CT complexation of π donors and acceptors. In contrast to hydrogen-bonded supramolecular assemblies,^20^ supramolecular stacks of donor–acceptor molecules are less extensively explored both in bulk and solution due to their comparatively low association constant.^19^ This typically results in self-sorting, which limits the formation of organized, multicomponent nanostructures held together by intermolecular D–A complexation.^21^

To arrive at hetero-aggregation between the D–A molecules, additional, supramolecular interactions are often included in the molecular design. Examples involve hydrogen-bonding,^22–24^ metal ion complexation,^25^ peptide-mediated assembly^26,27^ or amphiphilicity.^28,29^ Moreover, a geometrical fit between D and A improves the association since π-stacking is maximized by an increased contact area between the building blocks.^30,31^ Therefore, the acceptor naphthalene diimide (NDI) is often combined with the donor pyrene (Pyr). In crystals, this combination results in D–A CT dimers that stack well in the unit cell,^32,33^ while in polymeric and soft materials, the co-assembled packing of Pyr and NDI is much less ordered.^34^

Nanoscale order has been achieved in supramolecular polymers by phase segregation.^35^ Over the past decades, the covalent attachment of siloxanes to NDIs or pyrenes was studied thouroughly to obtain phase-segregated assemblies for *e.g.* self-healable and adaptive materials,^36–38^ or flexible devices.^39–41^ The highest degree of order is obtained for small molecule siloxane conjugates due to their discrete length. Recently, some of us pushed the boundaries of NDI–siloxane assemblies to a length scale between polymers and small molecules using discrete length oligodimethylsiloxanes (oDMS) end-capped with NDIs.^42^ The incompatibility of the oDMS and NDI parts in combination with crystallization of the NDIs yielded highly ordered lamellar morphologies with sub-10 nm domain sizes. The NDI–siloxane conjugates were utilized to pattern a graphite surface.^43^ Moreover, we have reported previously that conjugation of small molecules to discrete siloxanes can result in order over macroscopic length scales,^44,45^ giving unique properties to these siloxane-based materials.^46^

**Scheme 1:**
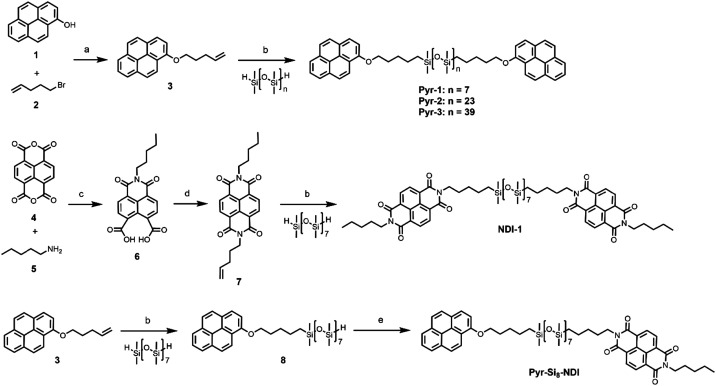
Synthesis of pyrene and NDI blocks and the corresponding Pyr-1, Pyr-2, Pyr-3, NDI-1 and Pyr-Si_8_-NDI. Reaction conditions: (a) K_2_CO_3_, KI, DMF, 80 °C, 24 h (89%); (b) Karstedt's catalyst, DCM, 1–2 h (51–83%); (c) DMF, microwave (i) 75 °C, 5 min, (ii) 140 °C, 15 min (22%); (d) 4-pentene-1-amine, DMF, microwave (i) 75 °C, 5 min, (ii) 140 °C, 15 min (48%); (e) NDI 7, Karstedt's catalyst, DCM, 4 h (45%).

Here, we report on the co-assembly of discrete NDI- and Pyr-oDMS block molecules to study the CT complexation of NDIs and pyrenes assisted by phase-segregation in bulk materials (Scheme 1). As a result of the discrete design and synergy between phase segregation and crystallization, we obtained crystalline order of the Pyr/NDI pairs when connecting the Pyr and NDI to the same siloxane oligomer forming a heterotelechelic oDMS. Hereby, we developed the first soft, CT material comprised of crystalline D–A dimers forming CT layers that are separated by an insulating layer. The robustness of the material and tuneability of the nanostructure are shown by high-pressure experiments, making this material relevant for soft nano-electronics.

## Results

### Synthesis of NDI- and Pyr-functionalized oDMS

We synthesized homotelechelic oligomers Pyr-1, Pyr-2, Pyr-3, and NDI-1, as well as a heterotelechelic oligomer denoted as Pyr-Si_8_-NDI. The pyrene-functionalized siloxanes were synthesized from 1-hydroxypyrene (1) and 5-bromo-1-pentene (2), forming the olefin-functionalized pyrene 3, which was used as the common building block for all Pyr-oDMS conjugates (Scheme 1). The oDMS dihydrides were obtained by a previously reported synthesis method,^47^ and used in the Karstedt's catalysed hydrosilylation reactions to obtain the final, coupled products. Pyrene 3 was coupled with oDMS-dihydrides with 8, 24 or 40 repeating units, resulting in Pyr-1, Pyr-2, and Pyr-3 (74–89%), respectively. NDI-1 was obtained from naphthalene tetracarboxylic dianhydride (4), *n*-pentylamine (5) and 4-penten-1-amine using a sequential microwave-assisted protocol (51%) (Scheme 1).^42^ Finally, the heterotelechelic siloxane (Pyr-Si_8_-NDI) with the pyrene on one chain-end and the NDI on the other end was synthesized from pyrene 3 and NDI 7. For this, a Karstedt catalysed hydrosilylation reaction was performed with one equivalent of the pyrene precursor and two equivalents of oDMS_8_ dihydride to prefer the formation of monofunctionalized siloxane (Scheme 1). However, due to the statistical nature of this reaction, a mixture of mono- and difunctionalized siloxane was obtained which was purified by column chromatography giving the mono-functionalized siloxane 8 in 56% yield. The remaining hydride functionality was coupled to NDI 7, yielding heterotelechelic Pyr-Si_8_-NDI (45%). All end-functionalized siloxanes were obtained in high purity and characterized as presented in the ESI‡ (Fig. S1–S5).

### Bulk co-assembly of homotelechelic NDI- and Pyr-oDMS

**Fig. 1:**
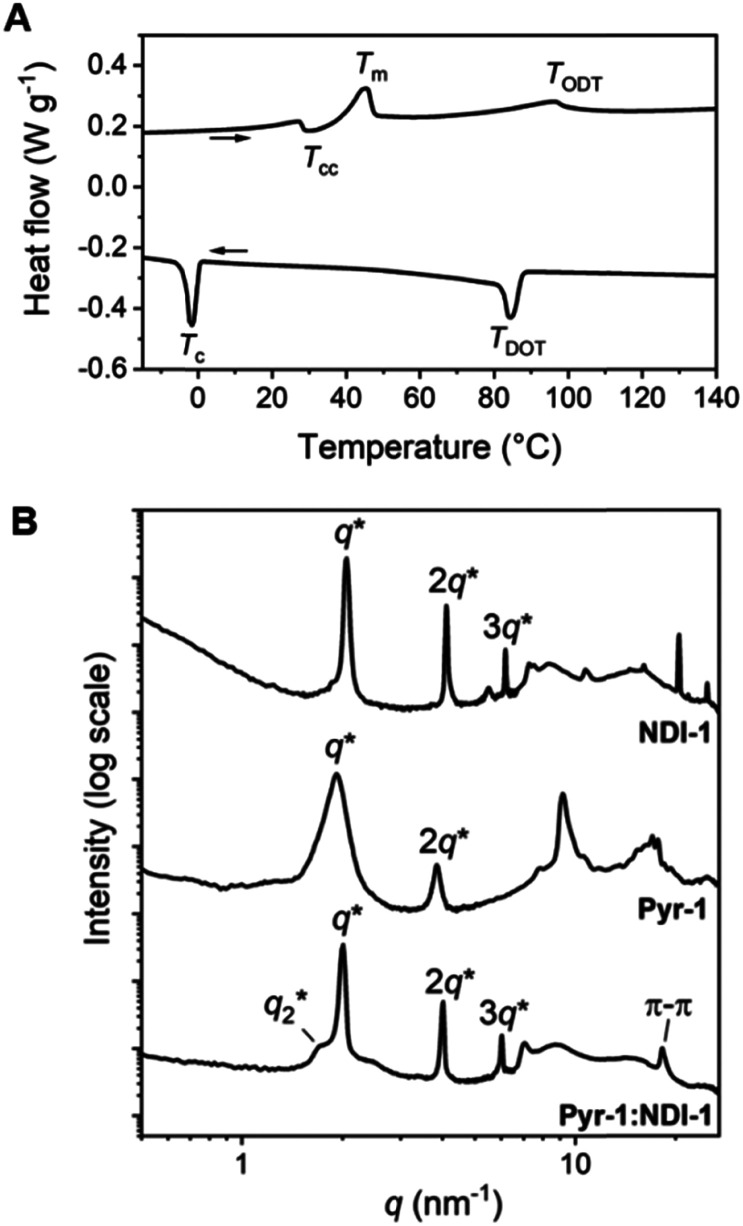
(A) DSC trace (second heating and cooling cycle) of Pyr-1:NDI-1. A temperature ramp of 10 K min^−1^ was used. Endothermic heat flows have a positive value. (B) 1D transmission scattering profiles of Pyr-1:NDI-1 (bottom), Pyr-1 (middle) and NDI-1 (top) at room temperature.

The three homotelechelic Pyr-oDMS block molecules were mixed with the NDI-1 block molecule to probe their CT properties and nanoscale organization. All mixtures were prepared in a 1 : 1 molar ratio by solvation in dichloromethane (DCM), followed by evaporation of the solvent. To exclude any solvent effects the material was molten at 120 °C and slowly cooled (5 K min^−1^) to room temperature. We here show the bulk co-assembly of Pyr-1 with NDI-1, denoted as Pyr-1:NDI-1 (Fig. 1). Similar results were obtained for mixtures Pyr-2:NDI-1 and Pyr-3:NDI-1 (Table 1) and are discussed in the ESI‡ (Fig. S7 and S8).

**Table 1:** Thermal properties and morphology of Pyr-oDMS and NDI-oDMS block molecules and mixtures thereof

Entry	Compound^a^	*f*_Si_ b	Appearance	*T*_m_ c (°C)	Δ*H*_fus_^c^ (kJ mol^−1^)	*T*_ODT_ c (°C)	*T*_DOT_ c (°C)	*T*_c_ c (°C)	*d* d (nm)
1	Pyr-1	0.59	Green solid	68.4	56.2	n.o.	n.o.	23.1	3.3
2	Pyr-2	0.81	Green liquid	19.5	57.4	n.o.	n.o.	11.0	6.2^e^
3	Pyr-3	0.88	Green liquid	6.0	50.6	n.o.	n.o.	0.1	7.9^f^
4	NDI-1	0.52	Off-white solid	160.6	17.9	n.o.	n.o.	142.8	3.1
5	Pyr-1:NDI-1	—	Purple wax	45.1	3.6^g^	95.6	84.1	−1.7	3.1
6	Pyr-2:NDI-1	—	Purple wax	n.o.	2.1^g^	80.3	75.2	n.o.	4.9
7	Pyr-3:NDI-1	—	Purple wax	n.o.	3.6^g^	70.3	63.1	n.o.	6.5
8	Pyr-Si_8_-NDI	0.55	Purple solid	92.2	10.9	n.o.	n.o.	87.6	3.1

a Block molecules and mixtures as depicted in Scheme 1.

b Volume fraction of siloxane (*f*_Si_) calculated from bulk densities of oDMS,^47^ NDI,^48^ and pyrene.^49^

c Melt transition temperature (*T*_m_) and the corresponding enthalpy of fusion per mole end-functionalized siloxane (Δ*H*_fus_), and order–disorder transition temperature (*T*_ODT_) measured using DSC, while heating at 10 K min^−1^. Disorder–order transition temperature (*T*_DOT_) and crystallization transition temperature (*T*_c_) measured using DSC, while cooling at 10 K min^−1^.

d Domain spacing of the lamellar structure, calculated using *d* = 2π/q*, obtained from SAXS at room temperature.

e Measured at 10 °C.

f Measured at −10 °C.

g Δ*H*_fus_ corresponding to *T*_ODT_. n.o. = not observed. DSC thermograms and SAXS profiles of all individual homotelechelic block molecules and mixtures can be found in the ESI (Fig. S6–S10).

The Pyr-1:NDI-1 mixture was obtained as a dark purple wax at room temperature, indicative of significant CT complexation. The formation of a CT complex was confirmed by UV-vis spectroscopy, showing a clear absorption band at 550 nm, typical for a Pyr–NDI D–A assembly (Fig. S9, ESI‡).^31^ The thermal analysis by differential scanning calorimetry (DSC) shows a cold crystallization temperature (*T*_cc_) followed by a melt transition temperature (*T*_m_) and a broad endothermic transition upon heating (Fig. 1A and Table 1 entry 5). We assign the latter to an order–disorder transition (*T*_ODT_) temperature as the enthalpic energy of the transition is relatively low (<3.6 kJ mol^−1^) (Table 1, entry 4). Upon cooling, a disorder–order transition temperature (*T*_DOT_) is observed at 84.1 °C, followed by a crystallization transition below room temperature (−1.7 °C). These results indicate the presence of a liquid crystalline phase at room temperature which is in accordance to the waxy appearance of the material. Moreover, birefringent textures were observed by polarized optical microscopy (POM) indicative of liquid crystalline ordering at room temperature (Fig. S7D, ESI‡).

We investigated the liquid crystalline order in the Pyr-1:NDI-1 mixture by medium- and wide-angle X-ray scattering (MAXS and WAXS) (Fig. 1B). The extent of mixing was determined by comparing the morphologies of the individual components Pyr-1 and NDI-1 with the mixture. A lamellar nanostructure is formed by both the Pyr-1 and NDI-1 block molecules as well as the mixture Pyr-1:NDI-1, indicated by the presence of q* and its integer multiples (Fig. 1B and Table 1). In the transmission scattering profile of Pyr-1:NDI-1, a small shoulder (*q*_2_*) next to the primary scattering peak q* is observed. The shoulder completely vanished at 60 °C upon heating, while it reappeared at 20 °C upon cooling and became even more pronounced at −20 °C (Fig. S10, ESI‡). This indicates that the *q*_2_* morphology is coupled to the thermal transitions *T*_m_ (45.1 °C) and *T*_c_ (−1.7 °C).

Multiple sharp scattering peaks were observed in the high-*q* region (*q* > 7 nm^−1^) for Pyr-1 and NDI-1, indicative of a highly crystalline structure (Fig. 1B). The Pyr-1:NDI-1 mixture is lacking these sharp reflection peaks, but a single peak is observed at 18.3 nm^−1^, representing a π-stacking distance of 0.34 nm. Hence, the crystalline order of the single components disappeared upon mixing in accordance with the DSC data. However, the Pyr-1:NDI-1 nanostructure is ordered by means of CT and π-stacking interactions in combination with nanophase segregation. From the presence of a single scattering profile at room temperature and the disparity from the individual components in the high-*q* region, we propose a co-assembled lamellar nanostructure for Pyr-1:NDI-1 in which the pyrenes and NDIs are randomly distributed in the Pyr/NDI layer. Similar results for the other mixtures are given in the ESI,‡ where also a short discussion in the influence of the siloxane length is given.

### Bulk co-assembly of Pyr-Si8-NDI

#### Improved CT properties and nanoscale order

To further improve the bulk co-assembly of the oDMS-functionalized pyrenes and NDIs, we attached both the donor and acceptor molecule in a 1 : 1 stoichiometry onto an oDMS of 8 repeating units, forming Pyr-Si_8_-NDI. As a result, the one-component Pyr-Si_8_-NDI block molecule is composed of identical components as the two-component Pyr-1:NDI-1 mixture.

**Fig. 2:**
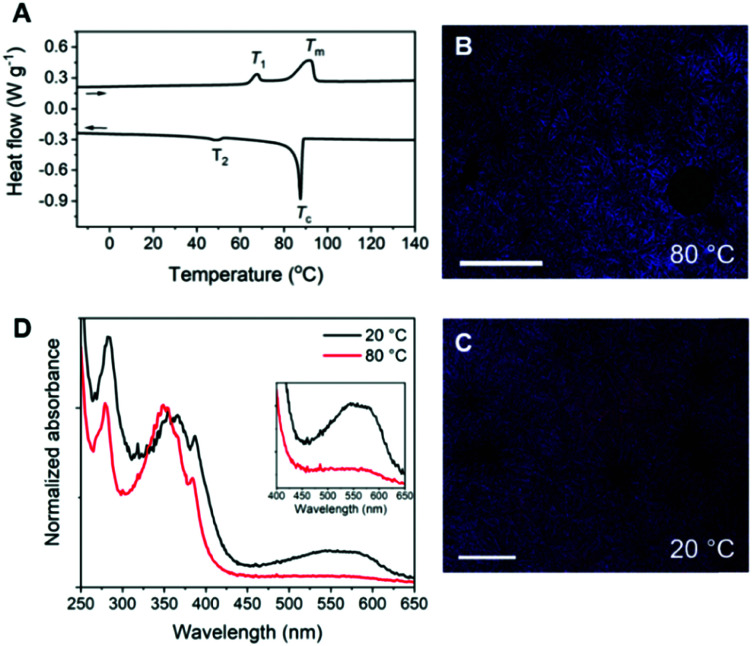
(A) DSC trace (second heating and cooling run) of Pyr-Si_8_-NDI. Endothermic heat flows have a positive value. A temperature ramp of 10 K min^−1^ was used. (B and C) POM images (crossed polarizers) of Pyr-Si_8_-NDI at (B) 80 °C and (C) 20 °C. The material was placed in between two glass slides, heated to the isotropic state and cooled with 5 K min^−1^ to room temperature. Scale bar represents 250 μm. (D) Normalized solid-state absorption spectra of Pyr-Si_8_-NDI, spin-coated (10 mg mL^−1^ in chloroform) on a quartz substrate, measured at 20 °C (black) and 80 °C (red). The inset shows the magnified absorption of the CT band.

The Pyr-Si_8_-NDI was obtained as a brittle, purple solid, indicative of CT complexation at room temperature. Two thermal transitions were observed both upon heating and cooling using DSC (Fig. 2A). Upon heating, the material passed through an endothermic transition (*T*_1_) at 67.3 °C with a relatively small enthalpic contribution (2.2 kJ mol^−1^), indicative of an order–order transition. This was followed by melting of the material at 92.2 °C (*T*_m_) with an energy release of 10.9 kJ mol^−1^. Subsequent cooling gave a crystallization transition temperature (*T*_c_) at 87.6 °C, followed by a weaker exothermic transition at 48.4 °C, which is assigned to an order–order transition (*T*_2_). The formation of small, birefringent spherulites was observed under the polarized optical microscope when a film of Pyr-Si_8_-NDI was cooled from the isotropic melt to 80 °C between two glass plates (Fig. 2B). The material is liquid crystalline in the temperature range between *T*_c_ and *T*_2_. Cooling further to room temperature resulted in a pink colour of the spherulites (Fig. 2C) indicating the formation of a crystalline CT complex. This was further confirmed by UV-vis spectroscopy measurements on a Pyr-Si_8_-NDI spin-coated film, which showed a CT band at 550 nm (Fig. 2D).^31^

**Fig. 3:**
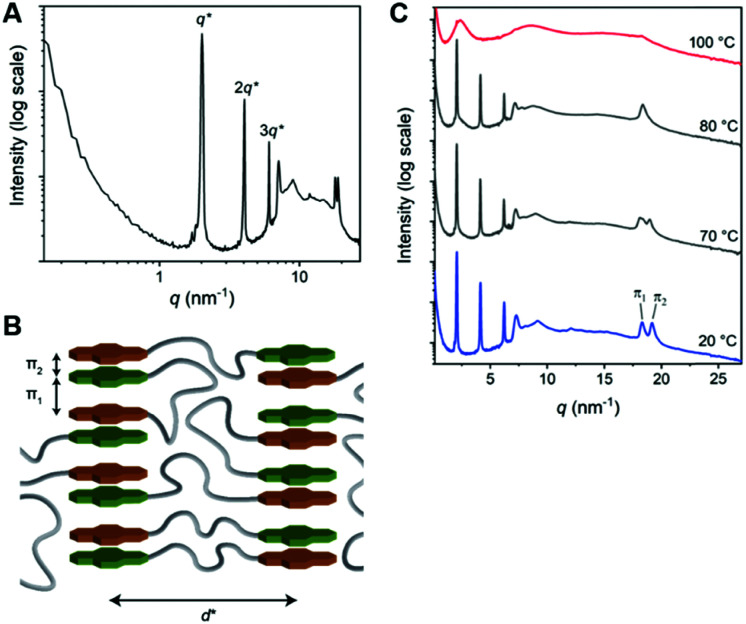
(A) 1D transmission scattering profile of Pyr-Si_8_-NDI at room temperature. (B) Schematic representation of the room temperature packing of Pyr-Si_8_-NDI. (C) Variable temperature 1D transmission scattering profile of Pyr-Si_8_-NDI upon heating.

The 1D transmission scattering profile of the Pyr-Si_8_-NDI bulk material shows very sharp scattering peaks at integer multiples of q* (Fig. 3A). This indicates the formation of a highly ordered lamellar nanostructure with a domain spacing of 3.1 nm. Crystallization of the CT complex is observed in the wide-angle region by the presence of sharp scattering peaks at *q* > 7 nm^−1^. Remarkably, two very sharp and high intensity peaks are present at 18.2 (π_1_) and 19.1 nm^−1^ (π_2_), representing distances of 0.35 and 0.33 nm, respectively. We attribute the distances of 0.33 (π_2_) and 0.35 (π_1_) nm to the π-stacking distance of a pyrene and NDI within a CT dimer and the distance between CT dimers, respectively.^32^ Therefore, we suggest that the pyrenes and NDIs form alternating stacks at room temperature, as schematically illustrated in Fig. 3B. This packing is similar to the packing of NDIs and pyrenes in CT co-crystals,^32^ but has not been observed for soft, polymeric or oligomeric CT materials to date. We expect that the high, crystalline order is due to the discrete design of the block molecules allowing for a perfect, defect free packing of the NDIs and pyrenes.

The π_1_ and π_2_ scattering peaks merged into one peak at 80 °C in the variable temperature transmission scattering data (Fig. 3C), representing a distance of 0.34 nm which is similar to the π-stacking of the Pyr-1:NDI-1 co-assembly. This, combined with the disappearance of the CT band in the absorption spectrum at 80 °C (Fig. 2D), strongly suggests that these distances are correlated with the CT complexation. We therefore propose that the CT dimers in Pyr-Si_8_-NDI vanish above *T*_1_ and that the NDIs and pyrenes distribute randomly throughout the layer due to the mobility in the liquid crystalline state (80 °C). As a result, the intensity of the CT band in the absorption spectrum strongly decreases, although the band is still present (Fig. 2D, inset). Thus, the liquid crystalline state shows less CT complexation than the crystalline state at room temperature. Upon cooling, the CT absorption band re-appears with the same intensity and the double π-stacking scattering peaks in the WAXS data emerge at 50 °C (Fig. S12, ESI‡) in accordance with the DSC data. This highlights the thermodynamic stability of the crystalline CT state formed by Pyr-Si_8_-NDI at room temperature.

#### Tuning the CT properties and domain spacing using pressure

**Fig. 4:**
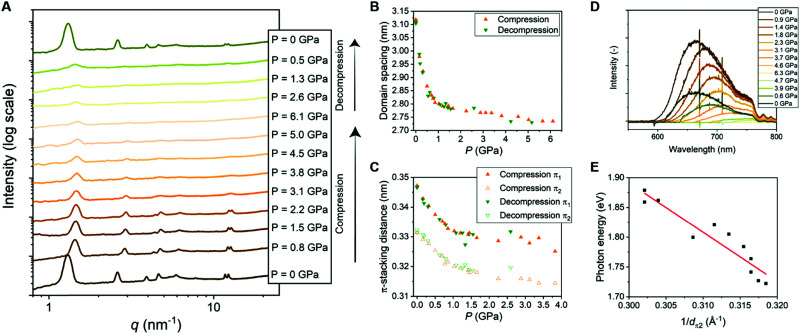
(A) 1D transmission scattering profiles of Pyr-Si_8_-NDI at room temperature under compression and decompression. (B) Domain spacing of the lamellar structure changes as a function of pressure. (C) π_1_- and π_2_-stacking distance changes as a function of pressure. (D) Emission spectra of Pyr-Si_8_-NDI (excited with 532 nm laser) at room temperature under compression and decompression. (E) Photon energy corresponding to the maximum intensity of the CT emission spectra for various pressures plotted against the inverse distance of π_2_ at the corresponding pressure. The red, fitted line indicates a linear relation (details in ESI‡).

Pressure-induced phase-transitions or structural changes are often reported for CT cocrystals.^7^ The presence of the Pyr/NDI CT cocrystals in the Pyr-Si_8_-NDI material raised curiosity regarding pressure-induced changes in the amorphous-crystalline soft material, which has not been reported before. We applied pressures up to 6.1 GPa to the material composed of isotropically ordered lamellae and probed the structural changes by wide angle X-ray scattering (WAXS) analysis (Fig. 4A). A rapid decrease in domain spacing of the lamellar structure was observed in the regime up to 1 GPa (Fig. 4B). Upon applying higher pressures, up to 6.1 GPa, the domain spacing slowly decreases further to 2.75 nm. Hence, the size of the nanostructure decreases with 12%, close to the compressibility of siloxanes which is 9%.^50^ Thus, the good compressibility of the siloxane linker might be the origin of the decrease in domain spacing, regardless of the material's crystalline nature. Both π-stacking distances (π_1_ and π_2_) – perpendicular to the lamellar domain spacing – also decrease upon applying pressure (Fig. 4C). Here, the decrease is only 6% for both π-stacking distances. We attribute the difference in compressibility of the Pyr/NDI stack and domain spacing to the crystalline and amorphous nature of the CT cocrystals and siloxane, respectively.

The π-stacking peaks broaden significantly and vanish at 4.5 GPa (Fig. 4A). The scattering peaks in the low-*q* regime, representing the lamellar packing, also broaden indicating a different order of the lamellar nanostructure. The peaks decrease in intensity, but never vanish and thereby the material comprises a nanophase-separated state that is stable up to 6.1 GPa. Upon decompression, all scattering peaks that are present before compression reappear at the same value of *q*, but with different intensities (Fig. 4A). The intensities from the transmission scattering profile as a function of pressure during compression and decompression indicate considerable hysteresis. Hence, full recovery of the lamellar, crystalline nanostructure with a high degree of order is only reobtained after full release of pressure to 0 GPa. In contrast, the domain spacing and π-stacking distances, that follow from *q*, increase without any hysteresis upon decompression (Fig. 4B and C).

The change in optical properties of Pyr-Si_8_-NDI upon applying pressure to the material was determined by fluorescence spectroscopy. The emission spectra – although of low intensity – were recorded at pressures up to 6.3 GPa (Fig. 4D). The peak maximum shifts from 660 nm at 0 GPa to higher wavelengths upon applying pressure (Fig. S13, ESI‡). The CT emission vanishes at 4.6 GPa indicated by the disappearance of the emission peak (Fig. 4D). The peak reappears upon decompression to 0.6 GPa and full decompression to 0 GPa shifts the peak maximum back to 660 nm. Hence, the intensity of the CT emission as a function of pressure indicates considerable hysteresis, similar to the hysteresis necessary to reobtain the highly ordered, lamellar packing (*vide supra*).

The red shift in CT emission upon applying pressure is indicative of lowering the energy of the CT state. To evaluate this hypothesis, we took the photon energy corresponding to the maximum in the CT emission spectrum at each pressure (*E*_CT_) (Fig. 4D) and plotted it against the corresponding inverse π_2_-stacking distance (1/*d*_π2_) (Fig. 4E). We find a linear correlation between the *E*_CT_ and 1/*d*_π2_. Thus, by applying pressure, one can tune the energy of the lowest excited state of a soft, CT material without losing its lamellar order. Future studies on electrical conductivity and optoelectronic functionality of this class of materials seem very promising.

## Discussion

### Pressure dependence of the energy of the charge transfer state in Pyr-Si8-NDI

We hypothesized that the red shift in charge transfer luminescence with increasing pressure is due to the closer proximity of the NDI and pyrene cores. The energy of the CT state may be approximated as *E*_CT_ = *E*_D/D^+^_ − *E*_A/A^−^_ + *E*_Coul_ with *E*_D/D^+^_ the oxidation potential of the donor, *E*_A/A^−^_ the reduction potential of the acceptor and *E*_Coul_ a term representing the coulombic electrostatic potential energy between the ionized donor D^+^ and acceptor A^−^ in the CT state. In the simplest possible description, one treats the ionized donor and acceptor as point charges so that *E*_Coul_ = −*q*_e_^2^/4π*ε*_0_*ε*_r_*d*_π2_ with *q*_e_ the electron charge, *ε*_0_ the permittivity of vacuum, *ε*_r_ the relative dielectric constant and *d*_π2_ the closest distance between donor and acceptor. If we assume *E*_D/D^+^_ and *E*_A/A^−^_ to be independent of applied pressure then the above expression for *E*_coul_ indeed accounts for the correlation observed in Fig. 4E between the *E*_CT_ state and 1/*d*_π2_. Moreover, assuming a value *ε*_r_ = 2 similar to benzene, one predicts a slope −*q*_e_^2^/4π*ε*_0_*ε*_r_ = −7.2 (eV Å) for the correlation between *E*_CT_ and 1/*d*_π2_, while from a fit to the experimental data in Fig. 4E we find a slope of −8 (eV Å). The value for the intercept in Fig. 4E amounts to 4.4 eV and might be interpreted as the energy of the CT state with the donor and acceptor moiety at infinite distance. The large difference between the estimated intercept (4 eV) and the energy of the CT state under ambient conditions (2 eV) indicates an exciton binding energy for the material in its lowest charge transfer excited state on the order of an eV. The close correspondence between experimental and predicted values for the slope of *E*_CT_*vs.* 1/*d*_π2_ supports our hypothesis that the redshift of fluorescence from the material at increased pressure is due to a reduction in the distance between Pyr and NDI moieties under compression.

In the above-described experiments, the pressure is applied on one side of the material and therefore we have to consider the direction of the lamellae in the material. The material is composed of isotropic ordered lamellar domains and therefore pressing in one direction of the material gives an average of the compression result. Pressing perpendicular to the lamellae affects mostly the distance between Pyr and NDI, while the siloxane is not much affected by the pressure in this direction. In contrast, pressing parallel to the lamellae compresses the siloxane significantly and the Pyr and NDI cores are not pushed together. As the material is composed of isotropically ordered lamellae, the lamellae exist in all angles towards the applied pressure direction. Therefore, average change in domain spacing, π-stacking distance, compressibility and fluorescence is obtained. Hence, future studies on Pyr-Si_8_-NDI with anisotropic lamellar domains are promising, allowing the tuning of the properties and distances in one direction instead of the average. In particular, lamellar domains that are oriented perpendicular to the pressing direction are highly interesting, as the Pyr and NDI molecules could possibly be pushed closer to each other and thereby lower the energy of the CT state even further.

### Differences in NDI/Pyr co-assemblies in one- *versus* two-component oDMS-based systems

The assembly morphology, CT properties, and stability of the CT state in the one-component Pyr-Si_8_-NDI and two-component Pyr-1:NDI-1 show striking differences. Therefore, we compare Pyr-Si_8_-NDI with Pyr-1:NDI-1, which are similar in terms of alkyl and siloxane linker lengths and stoichiometry of the D–A molecules, only the connectivity differs. In both cases, a lamellar nanostructure with a domain spacing of 3.1 nm was obtained. Nevertheless, two major differences in the CT material properties and nanostructure of the one-component Pyr-Si_8_-NDI*versus* the two-component Pyr-1:NDI-1 assemblies were observed. First, the highly efficient packing of the NDIs and pyrenes in the one-component system resulted in the formation of perfectly packed CT dimers at room temperature, while a more random distribution of pyrenes and NDIs within the stacks is observed for the two-component mixture. For the one-component Pyr-Si_8_-NDI system, we distinguish between the π-stacking distance of the CT dimers and within the dimer concluding a crystalline, alternate stacking of the pyrenes and NDIs which has not been reported for soft CT materials to date. In general, soft, non-crystalline CT materials show a single, broader π-stacking scattering peak at 0.34 nm,^34^ similar to the two-component Pyr-1:NDI-1 mixture at room temperature and Pyr-Si_8_-NDI above *T*_1_. The single π-stacking scattering peak indicates a random distribution of the NDIs and pyrenes in the CT stacks. Second, the CT nanostructure of Pyr-Si_8_-NDI is thermodynamically stable at room temperature as its order–order transition takes place at 48.8 °C. In contrast, the order–order transition of Pyr-1:NDI-1 is below room temperature (−1.7 °C). Hence, the two-component material is liquid crystalline at room temperature and therefore more fluctuations in the nanostructure are possible, while the one-component material is highly crystalline at room temperature.

Thereby, the Pyr-Si_8_-NDI system is able to endure high pressures while the two-component materials are waxes and therefore not suitable for pressure experiments. Nevertheless, the nanostructures of the two-component materials are readily changed by varying the length of one of the two components.

The amorphous oDMS linker, enabling movement of the pyrenes and NDIs in all directions, makes the categorization between inter- and intramolecular interactions – often investigated in solution^31^ – impossible in the bulk material. In particular, a pyrene in Pyr-Si_8_-NDI may interact with the NDI on the other end of the siloxane linker or with an NDI of another Pyr-Si_8_-NDI oligomer. We speculate that the difference in properties and packing between Pyr-Si_8_-NDI and Pyr-1:NDI-1 could be due to a larger number of assembly possibilities for the two-component Pyr-1:NDI-1 system which is a well-known phenomenon in supramolecular copolymers in solution.^51,52^ This makes the formation of a thermodynamically stable CT structure for Pyr-1:NDI-1 more demanding than for the heterotelechelic Pyr-Si_8_-NDI one-component system. Alternatively, the differences in CT properties for the one- and two-component assembly may arise from the highly effective molarity, potentially accessible with Pyr-Si_8_-NDI, as already shown in other supramolecular systems.^53^ Indeed, in solution we could observe this effect showing a clear CT band at 550 nm in the absorption spectrum of Pyr-Si_8_-NDI in methylcyclohexane (MCH), while no CT band was observed for the Pyr-1:NDI-1 mixture in MCH (Fig. S14, ESI‡). Hence, the pyrenes and NDIs co-assembled in the one-component Pyr-Si_8_-NDI system and self-sorted in the two-component Pyr-1:NDI-1 system in MCH. Interestingly, both one- and two-component systems formed a CT complex in oDMS solvent. These results are in analogy to the results by Mizuno and co-workers, showing CT complexation of pyrene and pyromellitic diimide in oDMS, while the co-assembly of the two molecules in aliphatic solvents was less successful.^54^ This highlights the importance of phase segregation induced by the siloxane for the co-assembly of the pyrenes and NDIs in solution and bulk.

## Conclusions

In conclusion, we successfully synthesized and assembled homo- and heterotelechelic, discrete siloxanes with pyrene (Pyr) and naphthalenediimide (NDI) peripheral blocks. We have shown that the covalent attachment of Pyr and NDI moieties by a siloxane linker in a heterotelechelic design influences the material properties and nanostructure significantly when compared to a homotelechelic, binary mixture. The co-assembly of homotelechelic Pyr- and NDI-oDMS resulted in a liquid crystalline material with a lamellar nanostructure in which the NDIs and pyrenes are randomly distributed throughout the Pyr/NDI layer. A highly ordered and thermodynamically stable CT material was formed by the heterotelechelic siloxane resulting from phase segregation induced by the siloxane, in synergy with co-crystallization of the NDI and pyrene. Herein, the NDIs and pyrenes form stacks characterized by crystalline CT dimers featuring an alternation of NDI and Pyr units. These are isolated in layers by the amorphous siloxane, forming a highly ordered lamellar nanostructure. The crystalline material is very robust and can undergo pressures up to 6.1 GPa at room temperature without losing the morphology. The NDI- and Pyr-cores are brought in closer proximity by pressure, resulting in charge delocalization which makes this Pyr-Si_8_-NDI system a promising material for semi-conducting purposes.

All together, we have shown the importance of the molecular design on the nanostructure and CT properties in a multicomponent, bulk assembly system. We were able to tune the Pyr–NDI interactions and nanostructure feature sizes by the molecular design and upon applying pressure. Thereby, we created a modular and robust system in which the distance between the CT layers and between the donor and acceptor molecules can be altered making these materials promising for soft nano-electronics.

## Author contributions

B. A. G. L., G. V., A. R. A. P., and E. W. M. conceived the project and directed the research. B. A. G. L., B. F. M. d. W., J. A. B., and F. V. d. G. conducted the synthesis, B. A. G. L., M. H. C. v. S., B. W. L. v. d. B. and F. V. d. G. characterized the morphologies, S. C. J. M. analyzed the optical measurements, K. K., H. S., and T. A. performed the high-pressure experiments, B. A. G. L. and E. W. M. wrote most of the paper and all authors contributed to the writing and editing of the final manuscript.

## Conflicts of interest

There are no conflicts to declare.

## Supplementary Material

MH-009-D1MH01141C-s001
