# Supplementary material for: Tuning the donor–acceptor interactions in phase-segregated block molecules
Source: Mater Horiz. 2021 Sep 20;9(1):294–302. doi: 10.1039/d1mh01141c (PMC8725796; doi:10.1039/d1mh01141c)
Supplement: MH-009-D1MH01141C-s001 [file MH-009-D1MH01141C-s001.pdf]

This journal is © The Royal Society of Chemistry 2021

Supplementary information

Tuning the donor-acceptor interactions in phase-segregated block molecules

Brigitte A.G. Lamers,[a] Martin H.C. van Son,[a] Freek V. de Graaf,[a] Bart W.L. van den Bersselaar,[a] Bas F.M. de Waal,[a] Kazuki Komatsu,[b] Hiroshi Sato,[c,d] Takuzo Aida,[c,e] José Augusto Berrocal,[f] Anja R.A. Palmans,[a] Ghislaine Vantomme,[a] Stefan C.J. Meskers,[g] and E.W. Meijer*[a]

[a.] Institute for Complex Molecular Systems and Laboratory of Macromolecular and Organic Chemistry, Eindhoven University of Technology, P.O. Box 513, 5600 MB Eindhoven, The Netherlands; Email: e.w.meijer@tue.nl.
[b.] Geochemistry Research Center, Graduate School of Science, The University of Tokyo, 7-3-1 Hongo, Bunkyo-ku, Tokyo 113-0033, Japan.
[c.] RIKEN Center for Emergent Matter Science, Wako, Saitama 351-0198, Japan
[d.] Japan Science and Technology Agency (JST), Precursory Research for Embryonic Science and Technology (PRESTO), 4-1-8 Honcho, Kawaguchi, Saitama 332-0012, Japan.
[e.] Department of Chemistry and Biotechnology, School of Engineering, The University of Tokyo, Bunkyo-ku, Tokyo 113-8656, Japan.
[f.] Adolphe Merkle Institute, Polymer Chemistry and Materials, University of Fribourg, Chemin des Verdiers 4, 1700 Fribourg, Switzerland.
[g.] Institute for Complex Molecular Systems and Molecular Materials and Nanosystems, Eindhoven University of Technology, P.O. Box 513, 5600 MB Eindhoven, The Netherlands.

Table of Contents

1. Materials and methods........S2
2. Synthetic procedures........S3
3. NMR spectra........S8
4. Bulk co-assembly of homotelechelic **NDI-1** with Pyr-*o*DMS, having a varying siloxane oligomer volume fraction........S13
5. Influence of Alkyl spacer on the morphology and CT properties of mixtures of **Pyr-1** with NDI block molecules........S16
6. Variable temperature X-ray measurements of **Pyr-Si$_8$-NDI**........S18
7. Changes in CT energy of **Pyr-Si$_8$-NDI** with pressure........S18
8. UV-Vis spectroscopy of **Pyr-1:NDI-1** and **Pyr-Si$_8$-NDI** in solution........S19
9. References........S19

1. Materials and Methods

All chemicals were purchased from commercial sources and used without further purification. The discrete length oligodimethylsiloxanes (*o*DMS) dihydride with a length of 8, 24 or 40 repeating units were synthesized according to literature procedure.[1] **NDI-3** and 10-undecen-1-amine were synthesized according to literature procedures.[2] Dry solvents were obtained with an MBRAUN solvent purification system (MB-SPS). Oven-dried glassware (120 °C) was used for all reactions carried out under argon atmosphere. Reactions were followed by thin-layer chromatography (TLC) using 60-F254 silica gel plates from Merck and visualized by UV light at 254 nm. Automated column chromatography was conducted on a Biotage Isolera One system using Biotage Sfär Silica Flash Cartridges.

NMR spectra were recorded on Bruker 400 MHz Ultrashield spectrometers (400 MHz for ^{1}H NMR, 100 MHz for ^{13}C NMR). Deuterated solvents used are indicated in each case. Chemical shifts (δ) are expressed in ppm and are referred to the residual peak of the solvent. Peak multiplicity is abbreviated as s: singlet; d: doublet, q: quartet; p: pentet; h: heptet; m: multiplet; dd: double doublet; dt: double triplet; ddt: double doublet of triplets.

Matrix assisted laser absorption/ionization-time of flight mass spectra (MALDI-ToF) were obtained on a Bruker Autoflex spectrometer using α-cyano-4-hydroxycinnamic acid (CHCA) or trans-2-[3-(4-tert-butylphenyl)-2-methyl-2-propenylidene]-malononitrile (DCBT) as matrix.

Polarized Optical Microscopy (POM) samples were placed on glass substrates and imaged using Nikon Xfinity1 Lumenera microscope with 5x magnification.

Differential scanning calorimetry (DSC) data were collected on a DSC Q2000 from TA instruments, calibrated with an indium standard. The samples (4-8 mg) were weighed directly into aluminium pans and hermetically sealed. The samples were initially heated to 180 °C and then subjected to two cooling/heating cycles from -50 °C to 180 °C with a rate of 10 K min^{-1}. The data that is presented, represents the second heating and/or cooling cycle.

Bulk small angle X-ray scattering (SAXS) was performed on an instrument from Ganesha Lab. The flight tube and sample holder are all under vacuum in a single housing, with a GeniX-Cu ultra low divergence X-ray generator. The source produces X-rays with a wavelength (λ) of 0.154 nm and a flux of 1 × 108 ph s^{-1}. Scattered X-rays were captured on a 2-dimensional Pilatus 300K detector with 487 × 619 pixel resolution. The sample-to-detector distance was 0.084 m (WAXS mode) or 0.48 m (MAXS mode). The instrument was calibrated with diffraction patterns from silver behenate.

High-pressure powder X-ray diffraction (HP-XRD) was performed using a Rigaku R-axisIV^{++} imaging plate diffractometer with a MicroMax-007 x-ray generator (MoKα, λ = 0.07107 nm) and a Varimax-Mo confocal mirror optics. The incident x-ray was collimated by a single pinhole colimator with a hole diameter of 0.3 mm. Powder crystal samples with small ruby balls as pressure markers were loaded into clamped diamond anvil cells made of cupper-bellyrium alloy without any pressure-transmitting medium. The load was controlled by high-pressure He gas via a membrane, or by 3 x M3 screws for higher load. A pair of Boehler-Almax type conical diamonds with 0.8 mm culet were used as anvils. Copper-bellyrium or Inconel alloy plates with a thickness of 0.1-0.2 mm and a hole diameter of 0.3-0.4 mm were used as gasket. Applied pressure was estimated by ruby fluorescence method.[3] The obtained 2D diffraction images were converted into 1D diffraction pattern using a IPAnalyzer and PDIndexer softwares.[4]

High-pressure fluorescence (HP-FL) measurements were performed on an OLYMPUS BX51 microscope. Samples were loaded in diamond anvil cells as well as HP-XRD. The samples were irradiated by a Laser Quantum Gem 532 DPSS laser (5mW on the stage of microscope), and the fluorescence was filtered by a Edmund optics 532 nm notch filter, and the spectra were obtained by an Ocean Optics USB2000 spectrometer. Exposure time was 0.1 sec, and the spectra were measured 5 times and then averaged.
Ultraviolet–visible (UV-vis) absorbance spectra were recorded on a Jasco V-650 UV/Vis spectrometer at 293 K with a Jasco ETCT-762 temperature controller. UV-vis measurements were performed using spincoated quartz plates or quartz cuvettes (1 cm) with a Teflon cap. For the thin film samples, the quartz substrates were cleaned by subsequent sonication of the substrate in ethanol and acetone for 10 minutes per solvent. The substrates were dried by a stream of air and the thin film samples were prepared by spincoating (800 rpm, 1 minute) 50 µL of a 10 mg/mL solution containing the material onto a cleaned quartz substrate. The solution samples were prepared by solvation of the material in the solvent. The samples prepared in *o*DMS_{15} solvent were heated to 80 °C and subsequently cooled to room temperature before the measurement.

2. Synthetic procedures

*1-(Pent-4-en-1-yloxy)pyrene (**3**)*

To a suspension of K_2CO_3 (1.60 g, 11.6 mmol, 2 eq) in dry DMF (0.2 M), 1-hydroxypyrene (**1**), 5-bromo-1-pentene (**2**) (1.73 g, 11.6 mmol, 2 eq) and KI (0.1 g, 0.56 mmol, 0.1 eq) were added. The reaction was stirred at 80 ˚C for 24 h. The reaction mixture was cooled to room temperature and 50 mL of acetone was added. The mixture was filtered by vacuum filtration using a Buchner funnel and paper filter. The filtrate was collected, and the acetone was removed by rotary evaporation. Subsequently, the crude mixture was precipitated in 300 mL water, dropwise and while stirring. The solids were isolated by filtration using vacuum filtration with a Buchner funnel and paper filter. The solid residue was dried in vacuo, yielding pure olefin functionalized pyrene **3** as a dark green solid (1.48 g, 89%). ^{1}H NMR (400 MHz, $CDCl_3$): δ = 8.50 (d, *J* = 9.2 Hz, 1H, Pyr-H), 8.16 – 8.02 (m, 4H, Pyr-H), 8.01 – 7.87 (m, 3H, Pyr-H), 7.52 (d, *J* = 8.4 Hz, 1H, CH-C-O), 5.96 (ddt, *J* = 16.9, 10.2, 6.6 Hz, 1H, CH=CH_2), 5.14 (m, 2H, CH=CH_2), 4.33 (t, *J* = 6.3 Hz, 2H, O-CH_2), 2.43 (q, *J* = 7.0 Hz, 2H, CH_2-CH=CH_2), 2.12 (p, *J* = 8.1 Hz, 2H, O-CH_2-CH_2); ^{13}C NMR (100 MHz, $CDCl_3$) δ = 153.23, 137.98, 131.88, 131.84, 127.38, 126.43, 126.19, 125.97, 125.93, 125.60, 125.11, 125.05, 124.33, 124.24, 121.39, 120.55, 115.47, 109.25, 68.23, 30.50, 28.83 ppm.

Pyr-1

Pyrene **3** (0.35 g, 1.2 mmol, 2.4 eq) and Si_8 dihydride (0.29 g, 0.5 mmol) were dissolved in DCM (1 mL). The reaction was stirred at room temperature for 2 h. After full conversion of the hydride, the crude mixture was purified by automated column chromatography using heptane/DCM (gradient 95/5 to 80/20) as eluent. The pure product **Pyr-Si_8-Pyr** was obtained as a dark-green solid (0.48 g, 83 %). ^{1}H NMR (400 MHz, $CDCl_3$) δ = 8.47 (d, *J* = 9.1 Hz, 2H, Pyr-H), 8.09 (dd, *J* = 8.4, 6.4 Hz, 6H, Pyr-H), 8.02 (d, *J* = 9.2 Hz, 2H, Pyr-H), 7.97 – 7.91 (m, 4H, Pyr-H), 7.87 (d, *J* = 9.0 Hz, 2H, Pyr-H), 7.52 (d, *J* = 8.4 Hz, 2H, CH-C-O), 4.31 (t, *J* = 7.2 Hz, 4H, O-CH_2), 2.01 (p, *J* = 6.6 Hz, 4H, O-CH_2-CH_2), 1.65 (p, *J* = 7.4 Hz, 4H, O-CH_2-CH_2-CH_2-CH_2), 1.54 – 1.44 (m, 4H, O-CH_2-CH_2-CH_2-CH_2), 0.69 – 0.58 (m, 4H, CH_2-Si$(CH_3)_2$), 0.11 – 0.05 (m, 48H, Si$(CH_3)_2$; ^{13}C NMR (100

MHz, $CDCl_3$) δ = 153.40, 131.90, 131.87, 127.40, 126.37, 126.17, 125.99, 125.62, 125.24, 125.13, 125.00, 124.29, 124.20, 121.47, 120.57, 109.26, 69.14, 32.04, 30.17, 29.45, 23.35, 22.85, 18.44, 14.27, 1.37, 1.26, 0.38, 0.15 ppm. MS (MALDI-ToF): *m/z* calc. for $C_{58}H_{86}O_9Si_8^+$: 1153.00; found 1151.44 Da $[M+H]^+$.

Pyr-2

Pyrene **3** (0.1 g, 0.35 mmol, 2.3 eq) and Si_{24} dihydride (0.26 g, 0.15 mmol) were dissolved in DCM (1 mL). The reaction was stirred at room temperature for 1 h. After full conversion of the hydride, the crude mixture was purified by automated column chromatography using heptane/DCM (gradient 95/5 to 80/20) as eluent. The pure product **Pyr-Si$_{24}$-Pyr** was obtained as a dark-green oil (0.26 g, 74 %). ^{1}H NMR (400 MHz, $CDCl_3$) δ = 8.47 (d, *J* = 9.1 Hz, 2H, Pyr-H), 8.09 (dd, *J* = 8.4, 6.4 Hz, 6H, Pyr-H), 8.03 (d, *J* = 9.2 Hz, 2H, Pyr-H), 7.98 – 7.92 (m, 4H, Pyr-H), 7.87 (d, *J* = 9.0 Hz, 2H, Pyr-H), 7.54 (d, *J* = 8.6 Hz, 2H, CH-C-O), 4.31 (t, *J* = 6.5 Hz, 4H, O-CH_2), 2.07 – 1.97 (m, 4H, O-CH_2-CH_2), 1.72 – 1.59 (m, 4H, O-CH_2-CH_2-CH_2-CH_2), 1.54 – 1.44 (m, 4H, O-CH_2-CH_2-CH_2-CH_2), 0.69 – 0.58 (m, 4H, CH_2-$Si(CH_3)_2$), 0.10 – 0.05 (m, 144H, $Si(CH_3)_2$); ^{13}C NMR (100 MHz, $CDCl_3$) δ = 153.26, 131.75, 127.25, 126.23, 126.03, 125.86, 125.47, 125.11, 125.00, 124.86, 124.14, 124.06, 121.33, 120.44, 109.12, 69.00, 30.02, 29.31, 23.21, 18.30, 1.42, 1.20, 1.11, 0.68, 0.23 ppm. MS (MALDI-ToF): *m/z* calc. for $C_{90}H_{182}O_{25}Si_{24}^+$: 2339.46; found 2337.38 Da $[M+H]^+$.

Pyr-3

Pyrene **3** (0.34 g, 1.2 mmol, 2.4 eq) and Si_{40} dihydride (0.29 g, 0.50 mmol) were dissolved in DCM (1 mL). The reaction was stirred at room temperature for 2 h. After full conversion of the hydride, the crude mixture was purified by automated column chromatography using heptane/DCM (gradient 95/5 to 80/20) as eluent. The pure product **Pyr-Si$_{40}$-Pyr** was obtained as a dark-green oil (0.48 g, 83 %). ^{1}H NMR (400 MHz, $CDCl_3$) δ = 8.47 (d, *J* = 9.1 Hz, 2H, Pyr-H), 8.09 (dd, *J* = 8.4, 6.4 Hz, 6H, Pyr-H), 8.03 (d, *J* = 9.2 Hz, 2H, Pyr-H), 7.98 – 7.93 (m, 4H, Pyr-H), 7.87 (d, *J* = 9.0 Hz, 2H, Pyr-H), 7.54 (d, *J* = 8.6 Hz, 2H, CH-C-O), 4.31 (t, *J* = 6.5 Hz, 4H, O-CH_2), 2.07 – 1.94 (m, 4H, O-CH_2-CH_2), 1.96 – 1.56 (m, 4H, O-CH_2-CH_2-CH_2-CH_2), 1.54 – 1.44 (m, 4H, O-CH_2-CH_2-CH_2-CH_2), 0.69 – 0.58 (m, 4H, CH_2-$Si(CH_3)_2$), 0.11 – 0.05 (m, 240H, $Si(CH_3)_2$); ^{13}C NMR (100 MHz, $CDCl_3$) δ = 153.26, 131.75, 127.41, 126.39, 126.19, 126.01, 125.63, 125.26, 125.15, 125.02, 124.30, 124.22, 121.49, 120.59, 109.27, 69.15, 30.19, 29.47, 23.37, 18.46, 1.57, 1.36, 1.20, 0.83, 0.38 ppm. MS (MALDI-ToF): *m/z* calc. for $C_{122}H_{374}O_{41}Si_{40}^+$: 3525.88; found 3524.05 Da $[M+H]^+$.

*1,3-Dioxo-2-pentyl-2,3-dihydro-1H-benzo[de]isoquinoline-6,7-dicarboxylic acid (**6**)*

NTCDA (**4**) (0.6 g, 2.24 mmol, 2 eq) and *n*-pentylamine (**5**) (0.10 g, 1.12 mmol) were loaded into a 20 mL microwave reaction vessel equipped with a magnetic stirrer. DMF (7 mL) was added, the vessel was sealed, and the suspension was sonicated for 15 minutes. The mixture was heated in the microwave (300 W) at 75 °C for 5 minutes, followed by heating at 140 °C for 15 minutes. The suspension was poured into 200 mL NaOH (1 M), precipitates formed, and the solids were removed by vacuum filtration using a Buchner funnel and paper filter. The filtrate was acidified with concentrated HCl (37%) until pH ~7. Then, the mixture was acidified to pH ~3 more gently using a 3M HCl solution resulting in the formation of precipitates. The mixture was filtered by vacuum filtration using a Buchner funnel and paper filter, and the solid residue was dried in vacuo at 80 °C overnight. Pure naphthalene mono-imide (NMI) **6** was obtained as a light-brown solid (86 mg, 22 %). ^{1}H NMR (400 MHz, DMSO-d_6): δ = 8.48 (d, *J* = 7.7 Hz, 2H,

HOOC-C-C$\underline{H}$), 8.03 (s, 2H, N-CO-C-C$\underline{H}$), 4.03 (t, *J* = 7.6 Hz, 2H, N-C$\underline{H}_2$-CH$_2$), 1.69 – 1.62 (m, 2H, N-CH$_2$-C$\underline{H}_2$), 1.38 – 1.30 (m, 4H, C$\underline{H}_2$-C$\underline{H}_2$-CH$_3$), 0.87 (t, *J* = 8.4 Hz, 3H, CH$_2$-C$\underline{H}_3$) ppm.

*2-(Pent-4-en-1-yl)-7-pentylbenzo[lmn][3,8]phenanthroline-1,3,6,8(2H,7H)-tetraone (**7**)*

NMI **6** (92.5 mg, 0.26 mmol) and 4-pentene-1-amine (34 mg, 0.39 mmol, 1.5 eq) were loaded into a 5 mL microwave reaction vessel equipped with a magnetic stirrer. DMF (4 mL) was added, the vessel was sealed and the suspension was sonicated for 10 minutes. The mixture was heated in the microwave (300 W) at 75 °C for 5 minutes, followed by heating at 140 °C for 15 minutes. The suspension was poured into 100 mL water, precipitates formed, and the solids were isolated by vacuum filtration using a Buchner funnel and paper filter. The solids were washed thoroughly with water. Subsequently, the solid residue was dried in vacuo at 100 °C overnight. The crude product was dissolved in $CHCl_3$, filtered over a silica plug (4 cm) and eluted with $CHCl_3$. The product was dried in vacuo, yielding pure NDI **7** as a light pink solid (50 mg, 48%). ^{1}H NMR (400 MHz, $CDCl_3$): δ = 8.76 (s, 4H, Ar-$\underline{H}$), 5.90 (ddt, *J* = 16.7, 10.0, 6.7 Hz, 1H, C$\underline{H}$=CH$_2$), 5.16 – 4.96 (m, 2H, CH=C$\underline{H}_2$), 4.32 – 4.14 (m, 4H, N-C$\underline{H}_2$), 2.22 (q, *J* = 7.1 Hz, 2H, C$\underline{H}_2$-CH=CH$_2$), 1.89 (p, *J* = 7.6 Hz, 2H, C$\underline{H}_2$-CH$_2$-CH=CH$_2$), 1.75 (t, *J* = 7.4 Hz, 2H, C$\underline{H}_2$-CH$_2$-CH$_2$-CH$_3$), 1.47 – 1.38 (m, 4H, CH$_2$-(C$\underline{H}_2$)$_2$-CH$_3$), 0.93 (t, *J* = 6.9 Hz, 3H, CH$_2$-C$\underline{H}_3$); ^{13}C NMR (100 MHz, $CDCl_3$) δ = 162.98, 162.96, 137.59, 131.09, 131.07, 126.84, 126.82, 126.78, 126.72, 115.40, 41.11, 40.67, 31.34, 29.32, 27.90, 27.19, 22.54, 14.11 ppm. LC-MS: *m/z* calc. for $C_{24}H_{24}N_2O_2^+$: 405.47; found 405.17 Da [M+H]$^+$.

NDI-1

NDI **7** (38 mg, 0.09 mmol, 2.2 eq) and Si$_8$ dihydride (25 mg, 0.04 mmol) were dissolved in DCM (1 mL). The reaction was stirred at room temperature for 1 h. After full conversion of the hydride, the crude mixture was purified by automated column chromatography using heptane/DCM (gradient 50/50 to 0/100) as eluent. The pure product **NDI-1** was obtained as an off-white solid (30 mg, 51 %). ^{1}H NMR (400 MHz, $CDCl_3$) δ = 8.74 (s, 8H, NDI-$\underline{H}$), 4.18 (t, *J* = 7.5, 8H, N-C$\underline{H}_2$-CH$_2$), 1.73 (p, *J* = 8.1 Hz, 8H, N-CH$_2$-C$\underline{H}_2$), 1.51 – 1.33 (m, 16H, N-CH$_2$-CH$_2$-C$\underline{H}_2$-C$\underline{H}_2$), 0.92 (d, *J* = 6.7 Hz, 6H, CH$_2$-CH$_2$-$\underline{CH_3}$), 0.59 – 0.53 (m, 4H, C$\underline{H}_2$-Si(CH$_3$)$_2$), 0.10 – 0.01 (m, 60H, Si(C$\underline{H}_3$)$_2$); ^{13}C NMR (100 MHz, $CDCl_3$) δ = 162.87, 162.83, 130.88, 126.72, 126.70, 126.67, 53.59, 40.96, 30.85, 29.24, 27.85, 27.79, 23.05, 22.45, 18.21, 13.97, 1.16, 1.06, 0.14 ppm. MS (MALDI-ToF): *m/z* calc. for $C_{64}H_{98}N_4O_{15}Si_8Na^+$: 1409.49; found 1409.55 Da [M+Na]$^+$.

*Pyr-Si$_8$-hydride (**8**)*

Pyrene **3** (0.08 g, 0.28 mmol) and Si$_8$ dihydride (0.32 g, 0.56 mmol, 2 eq) were dissolved in DCM (1 mL). The reaction was stirred at room temperature for 1 h. After full conversion of the olefin, the crude mixture was purified by automated column chromatography using heptane/DCM (gradient 90/10 to 70/30) as eluent. The pure product Pyr-Si$_8$-H (**8**) was obtained as a green oil (0.13 g, 56 %). ^{1}H NMR (400 MHz, $CDCl_3$) δ = 8.49 (d, *J* = 9.2 Hz, 1H, Pyr-$\underline{H}$), 8.14 – 8.07 (m, 3H, Pyr-$\underline{H}$), 8.04 (d, *J* = 9.2 Hz, 1H, Pyr-$\underline{H}$), 7.99 – 7.93 (m, 2H, Pyr-$\underline{H}$), 7.88 (d, *J* = 8.9 Hz, 1H, Pyr-$\underline{H}$), 7.54 (d, *J* = 8.4 Hz, 1H, C$\underline{H}$-C-O), 4.72 (h, *J* = 2.8 Hz, 1H, Si(CH$_3$)$_2$-$\underline{H}$), 4.33 (t, *J* = 6.5 Hz, 2H, O-C$\underline{H}_2$), 2.02 (p, *J* = 6.7 Hz, 2H, O-CH$_2$-C$\underline{H}_2$), 1.67 (p, *J* = 7.2 Hz , 2H, O-CH$_2$-CH$_2$-CH$_2$-C$\underline{H}_2$), 1.53 – 1.46 (m, 2H, O-CH$_2$-CH$_2$-C$\underline{H}_2$-CH$_2$), 0.72 – 0.60 (m, 2H, C$\underline{H}_2$-Si(CH$_3$)$_2$), 0.20 (d, *J* = 2.8 Hz, 6H, Si(C$\underline{H}_3$)$_2$-H), 0.15 – 0.04 (m, 42H, Si(C$\underline{H}_3$)$_2$); ^{13}C NMR (100 MHz, $CDCl_3$) δ = 153.41, 131.90, 127.41, 126.38, 126.18, 126.01, 125.63, 125.26, 125.16, 125.01, 124.26, 121.49, 120.59, 109.26, 69.14, 30.19, 29.47, 23.37,

18.47, 1.38, 1.24, 1.03, 0.86, 0.40 ppm. MS (MALDI-ToF): *m/z* calc. for $C_{37}H_{68}O_8Si_8^+$: 864.31; found 864.33 Da $[M+H]^+$.

Pyr-Si$_8$-NDI

Pyr-Si_8-hydride (**8**) (23 mg, 0.03 mmol) and NDI (**7**) (12 mg, 0.03 mmol, 1.1 eq) were dissolved in DCM (1 mL). The reaction was stirred at room temperature for 1 h. After full conversion of the hydride, the crude mixture was purified by automated column chromatography using heptane/DCM (gradient 90/10 to 80/20) as eluent. The pure product **Pyr-Si$_8$-NDI** was obtained as a purple solid (24 mg, 45 %). ^1H NMR (400 MHz, $CDCl_3$) δ = 8.42 (s, 4H, NDI-H), 8.27 (d, *J* = 8.9 Hz, 1H, Pyr-H), 7.99 – 7.91 (m, 3H, Pyr-H), 7.90 – 7.81 (m, 2H, Pyr-H), 7.79 – 7.75 (m, 1H, Pyr-H), 7.73 – 7.69 (m 1H, Pyr-H), 7.43 (d, *J* = 8.4 Hz, 1H, Pyr-H), 4.29 (t, *J* = 6.4 Hz, 2H, Pyr-O-CH_2), 4.15 (t, *J* = 7.7 Hz, 4H, N-CH_2), 2.02 (q, *J* = 7.1 Hz, 2H, O-CH_2-CH_2), 1.80 – 1.72 (m, 4H, N-CH_2-CH_2), 1.72 – 1.63 (m, 2H, O-CH_2-CH_2-CH_2-CH_2), 1.52 – 1.36 (m, 10H, CH_2-CH_2-CH_2-Si_7-CH_2-CH_2-CH_2), 1.29 – 1.22 (m, 2H, CH_2-CH_3), 0.99 – 0.91 (m, 3H, CH_2-CH_3), 0.70 – 0.62 (m, 2H, O-$(CH_2)_4$-CH_2-$Si(CH_3)_2$), 0.62 – 0.55 ($Si(CH_3)_2$-CH_2-$(CH_2)_4$-N), 0.14 – 0.03 (m, 48H, $Si(CH_3)_2$); ^{13}C NMR (100 MHz, $CDCl_3$) δ = 162.96, 162.90, 153.38, 131.81, 131,78, 130.85, 129.64, 127.36, 126.75, 126.48, 126.33, 126.19, 125.62, 125.13, 124.97, 124.25, 121.44, 120.45, 109.20, 69.12, 41.09, 30.97, 30.19, 29.41, 27.95, 23.27, 22.57, 18.41, 14.13, 1.57, 1.35, 1.20, 0.83, 0.35. MS (MALDI-TOF): *m/z* calc. for $C_{61}H_{92}N_2O_{12}Si_8^+$: 1268.48; found 1269.50 Da $[M+H]^+$.

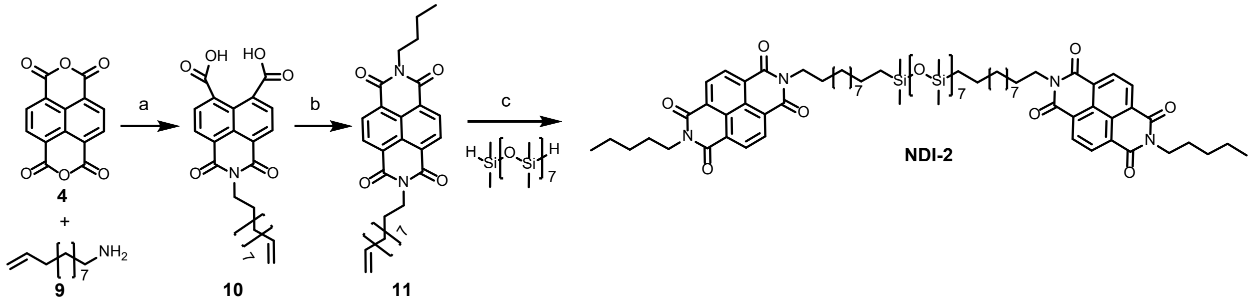

Scheme S1. Synthesis of the reference molecule **NDI-2**. Reaction conditions: (a) DMF, microwave i) 75 °C, 5 min, ii) 140 °C, 15 min (57%); (b) *n*-pentylamine, DMF, microwave i) 75 °C, 5 min, ii) 140 °C, 15 min (28%); (c) Karstedt's catalyst, DCM, r.t., 1 h (20%).

*1,3-Dioxo-2-(undec-10-en-1-yl)-2,3-dihydro-1H-benzo[de]isoquinoline-6,7-dicarboxylic acid (**10**)*

NTCDA (**4**) (1.35 g, 2.24 mmol, 2.5 eq) and 10-undecen-1-amine (**9**) (0.34 g, 2.01 mmol) were loaded into a 20 mL microwave reaction vessel equipped with a magnetic stirrer. DMF (7 mL) was added, the vessel was sealed, and the suspension was sonicated for 15 minutes. The mixture was heated in the microwave (300 W) at 75 °C for 5 minutes, followed by heating at 140 °C for 15 minutes. The suspension was poured into 150 mL NaOH (1 M), precipitates formed, and the mixture was filtered using a paper filter. The filtrate was acidified with 3 M HCl until pH ~5 was reached and precipitates formed. The mixture was filtered by vacuum filtration using a Buchner funnel and paper filter, and the residue was dried in vacuo at 80 °C overnight. Pure naphthalene mono-imide (NMI) **10** was obtained as a light-brown solid (500 mg, 57%). ^1H NMR (400 MHz, DMSO-d_6): δ = 8.48 (d, *J* = 7.5 Hz, 2H, HOOC-C-CH), 8.03 (d, *J* = 7.7 Hz, 2H, N-CO-C-CH),

5.77 (ddt, *J* = 16.6, 8.4 Hz, 1H, $C\underline{H}=CH_2$), 5.11 – 4.84 (m, 2H, $CH=C\underline{H}_2$), 4.03 (t, *J* = 7.3 Hz, 2H, $N\text{-}C\underline{H}_2\text{-}CH_2$), 1.99 (q, *J* = 8.0 Hz, 2H, $C\underline{H}_2\text{-}CH=CH_2$), 1.68 – 1.57 (m, 2H, $N\text{-}CH_2\text{-}C\underline{H}_2$), 1.38 – 1.10 (m, 12H, $CH_2\text{-}(C\underline{H}_2)_6$).

*2-Pentyl-7-(undec-10-en-1-yl)benzo[lmn][3,8]phenanthroline-1,3,6,8(2H,7H)-tetraone (**11**)*

NMI **10** (0.5 g, 1.14 mmol) and *n*-pentylamine (0.5 g, 5.71 mmol, 5 eq) were loaded into a 5 mL microwave reaction vessel equipped with a magnetic stirrer. DMF (4 mL) was added, the vessel was sealed, and the suspension was sonicated for 10 minutes. The mixture was heated in the microwave (300 W) at 75 °C for 5 minutes, followed by heating at 140 °C for 15 minutes. The suspension was poured into 100 mL water, precipitates formed, and the mixture was filtered under vacuum using a Buchner funnel and paper filter. The filter cake was washed with water and dried in vacuo at 100 °C overnight. The crude product was dissolved in DCM, filtered over a silica plug (4 cm) and eluted with DCM. The solvent was removed in vacuo and pure NDI **11** was obtained as a light pink solid (130 mg, 28%). ^1H NMR (400 MHz, $CDCl_3$): δ = 8.74 (s, 4H, Ar-$\underline{H}$), 5.80 (ddt, *J* = 16.9, 9.9, 6.7 Hz, 1H, $C\underline{H}=CH_2$), 5.02 – 4.82 (m, 2H, $CH=C\underline{H}_2$), 4.18 (t, *J* = 7.5 Hz, 4H, $N\text{-}C\underline{H}_2$), 2.02 (q, *J* = 6.8 Hz, 2H, $C\underline{H}_2\text{-}CH=CH_2$), 1.78 – 1.69 (m, 4H, $N\text{-}CH_2\text{-}C\underline{H}_2$), 1.44 – 1.20 (m, 20H, $CH_2\text{-}(C\underline{H}_2)_8\text{-}CH_3$ and $CH_2\text{-}(C\underline{H}_2)_2\text{-}CH$), 0.98 – 0.88 (m, 3H, $CH_2\text{-}C\underline{H}_3$) ppm.

NDI-2

NDI **11** (130 mg, 0.27 mmol, 2.6 eq) and Si_8 dihydride[1] (59 mg, 0.10 mmol) were dissolved in DCM (1 mL). The reaction was stirred at room temperature for 1 hour. After full conversion of the hydride, the crude mixture was purified by automated column chromatography using heptane/DCM (gradient 80/20 to 0/100) as eluent. The pure product **NDI-2** was obtained as an off-white solid (30 mg, 20%). ^1H NMR (400 MHz, $CDCl_3$) δ = 8.74 (s, 8H, NDI-$\underline{H}$), 4.18 (t, *J* = 7.4, 8H, $N\text{-}C\underline{H}_2\text{-}CH_2$), 1.72 (t, *J* = 10.1 Hz, 8H, $N\text{-}CH_2\text{-}C\underline{H}_2$), 1.46 – 1.18 (m, 20H, $CH_2\text{-}(C\underline{H}_2)_2\text{-}CH_3$ and $CH_2\text{-}(C\underline{H}_2)_8\text{-}CH_2\text{-}Si(CH_3)_2$), 0.92 (t, *J* = 6.5 Hz, 6H, $CH_2\text{-}\underline{CH_3}$), 0.54 – 0.47 (m, 4H, $C\underline{H}_2\text{-}Si(CH_3)_2$), 0.11 – 0.01 (m, 60H, $Si(C\underline{H}_3)_2$); ^{13}C NMR (100 MHz, $CDCl_3$) δ = 162.98, 162.96, 131.06, 126.83, 126.79, 41.15, 41.10, 33.63, 29.81, 29.75, 29.71, 29.56, 29.50, 29.32, 28.25, 27.90, 27.26, 23.38, 22.54, 18.42, 14.10, 1.32, 1.22, 1.14, 0.46, 0.34, 0.13. MS (MALDI-ToF): *m/z* calc. for $C_{76}H_{122}N_4O_{15}Si_8^+$: 1554.91 Da $[M+H]^+$; found 1554.98 Da.

3. NMR spectra

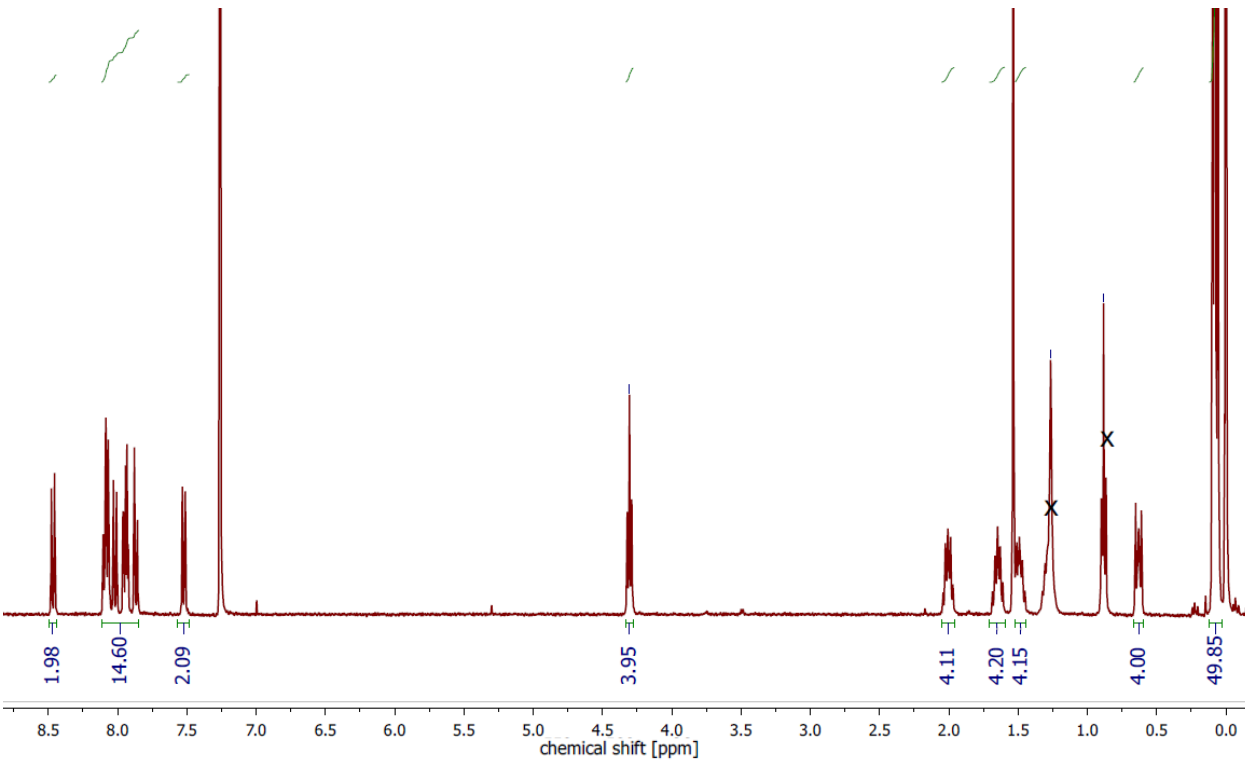

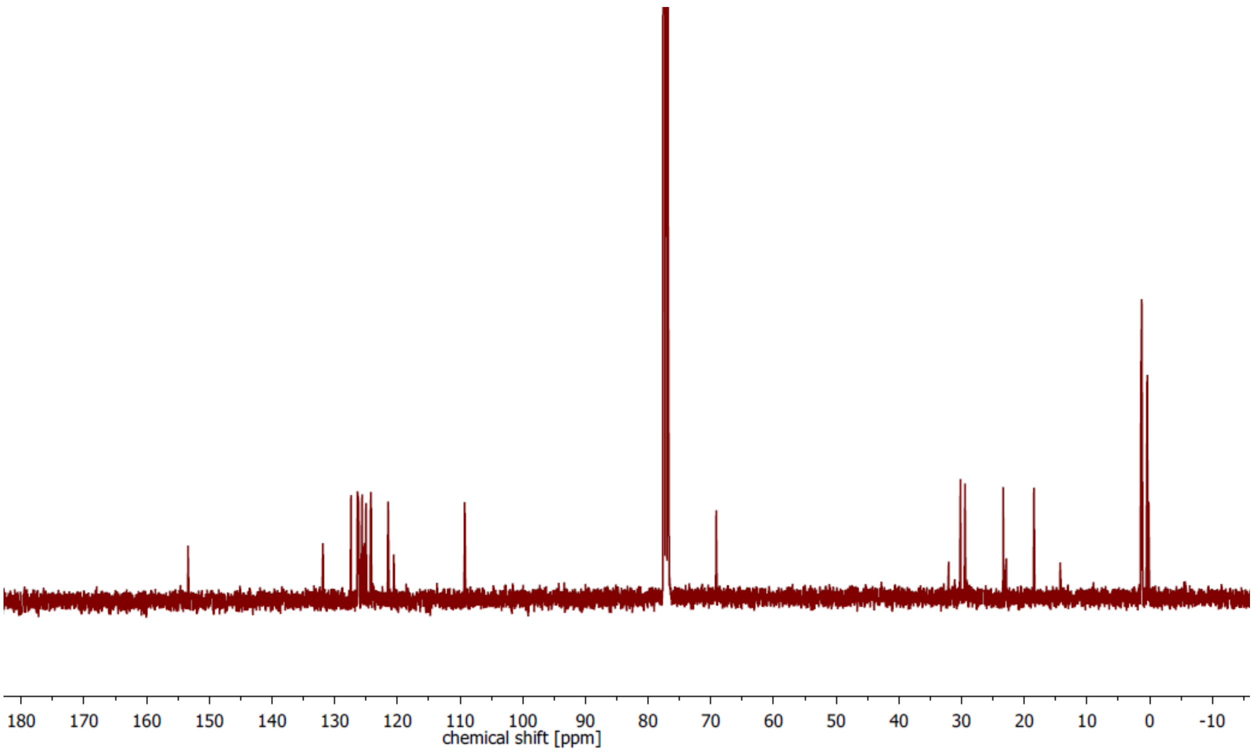

Figure S1. ^{1}H and ^{13}C NMR of **Pyr-1** ($CDCl_3$)

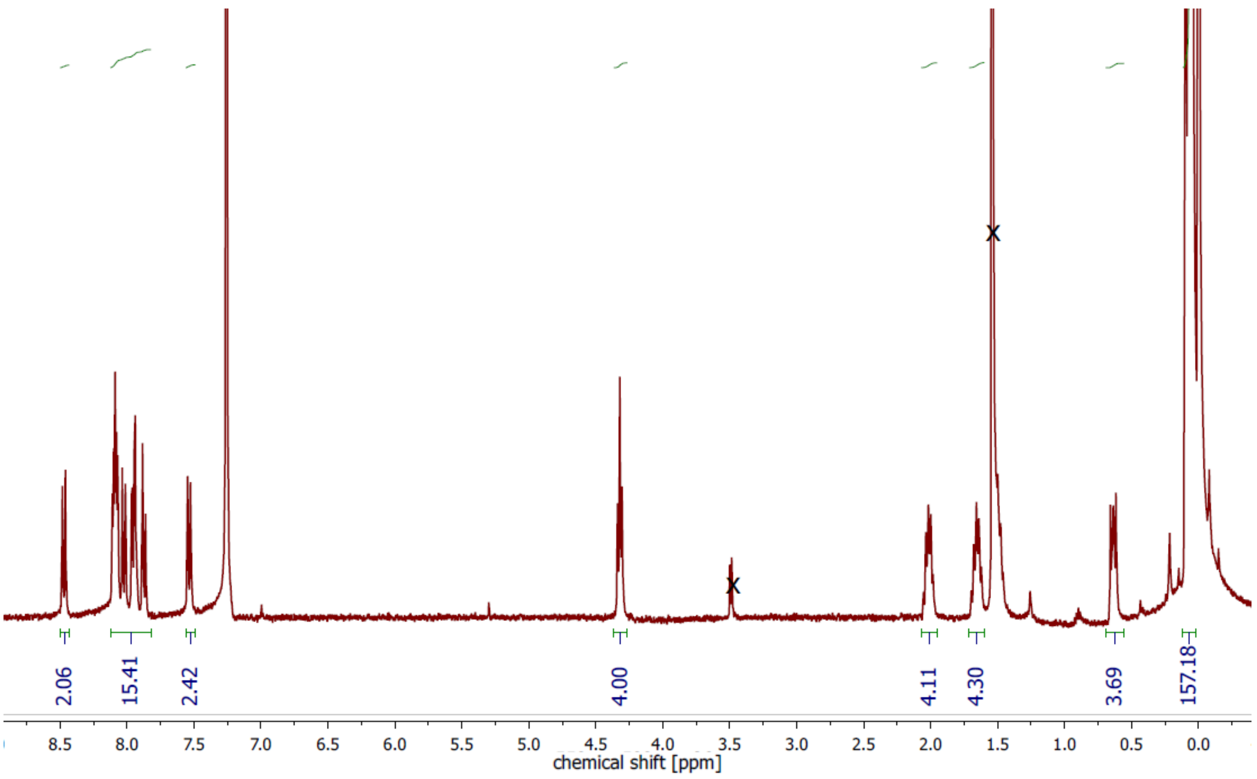

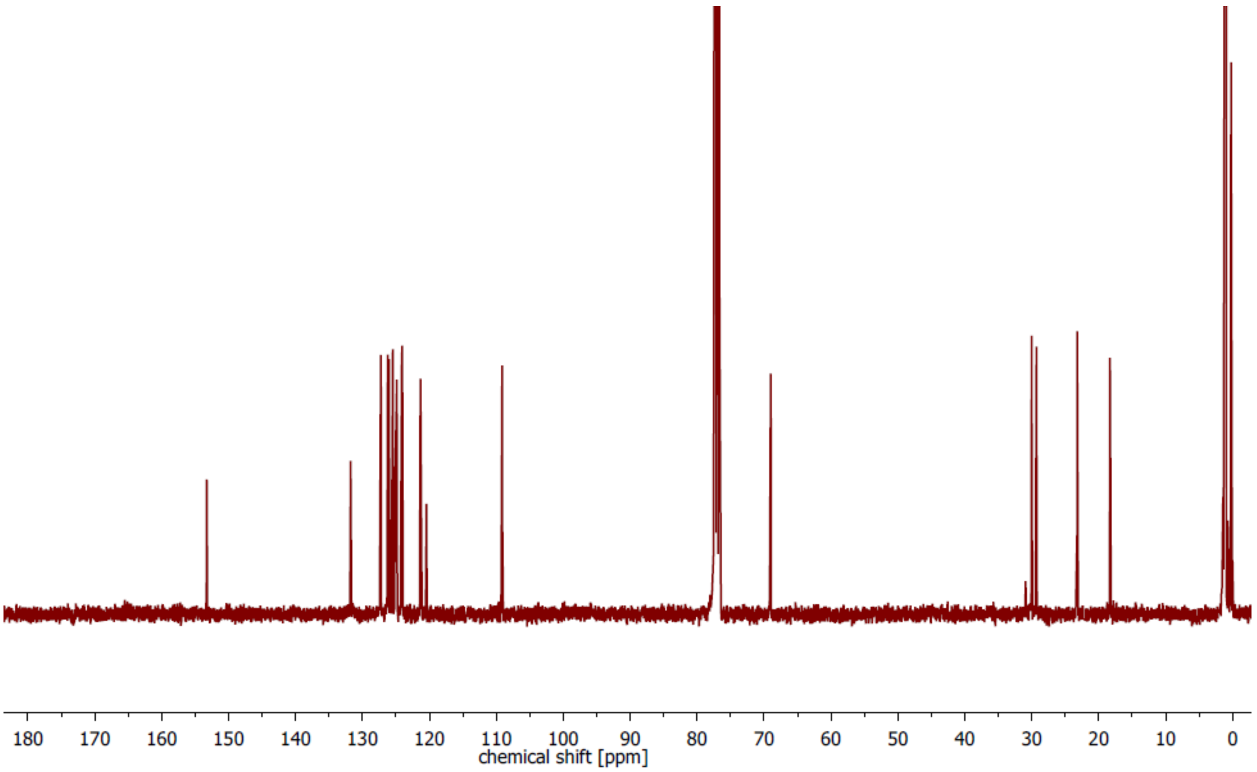

Figure S2. ^{1}H and ^{13}C NMR of **Pyr-2** ($CDCl_3$)

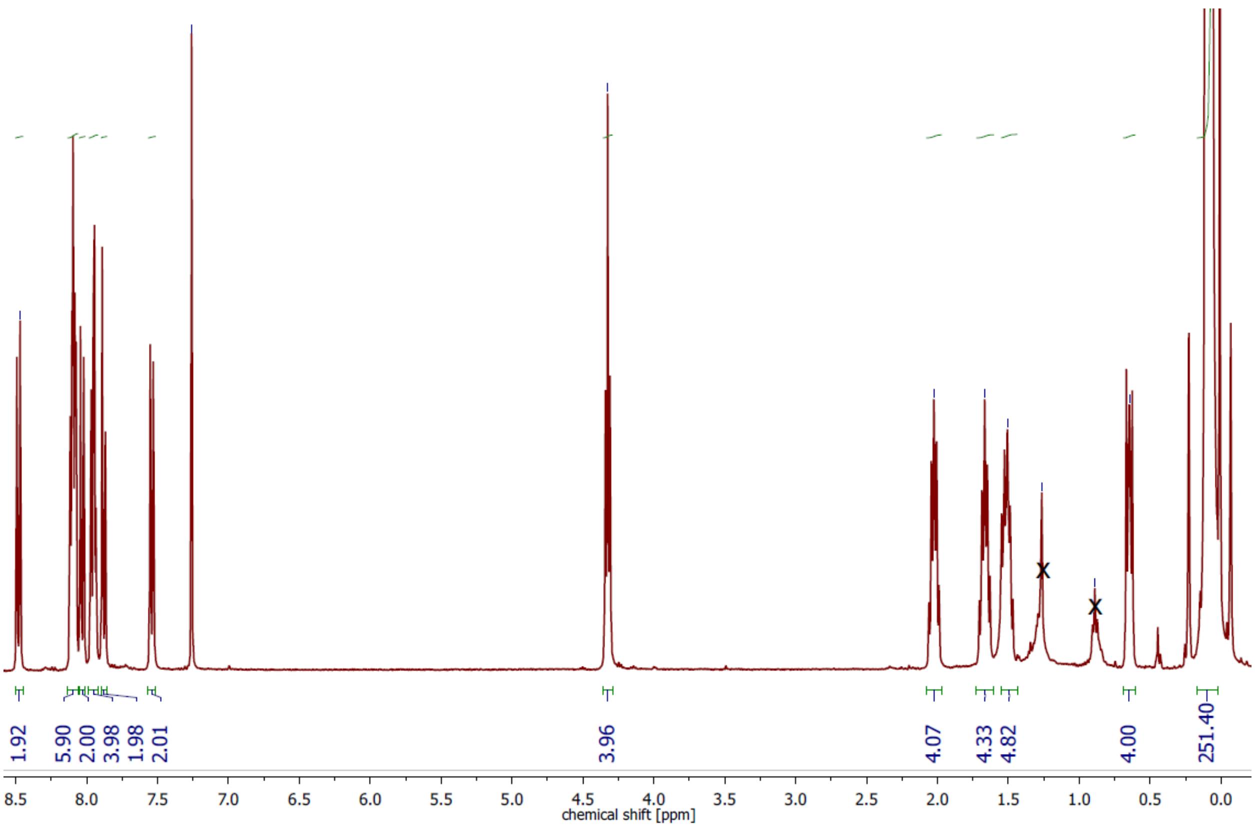

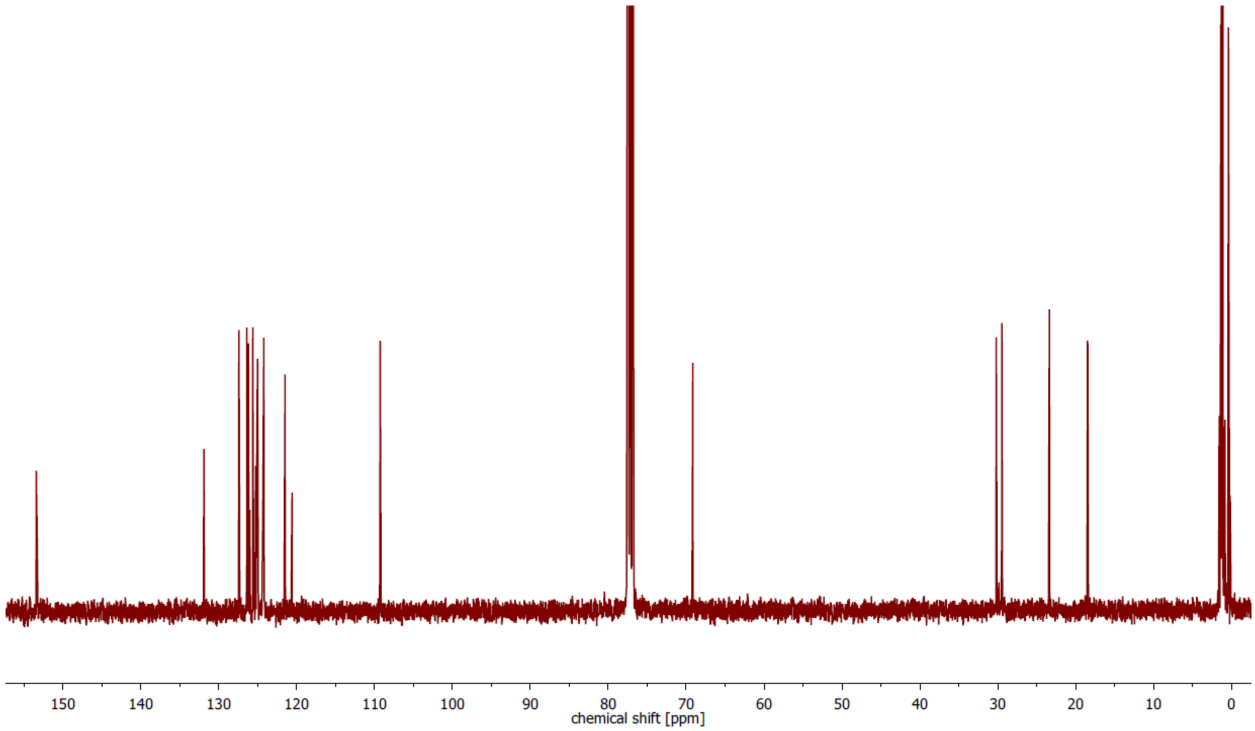

Figure S3. ^{1}H and ^{13}C NMR of **Pyr-3** ($CDCl_3$)

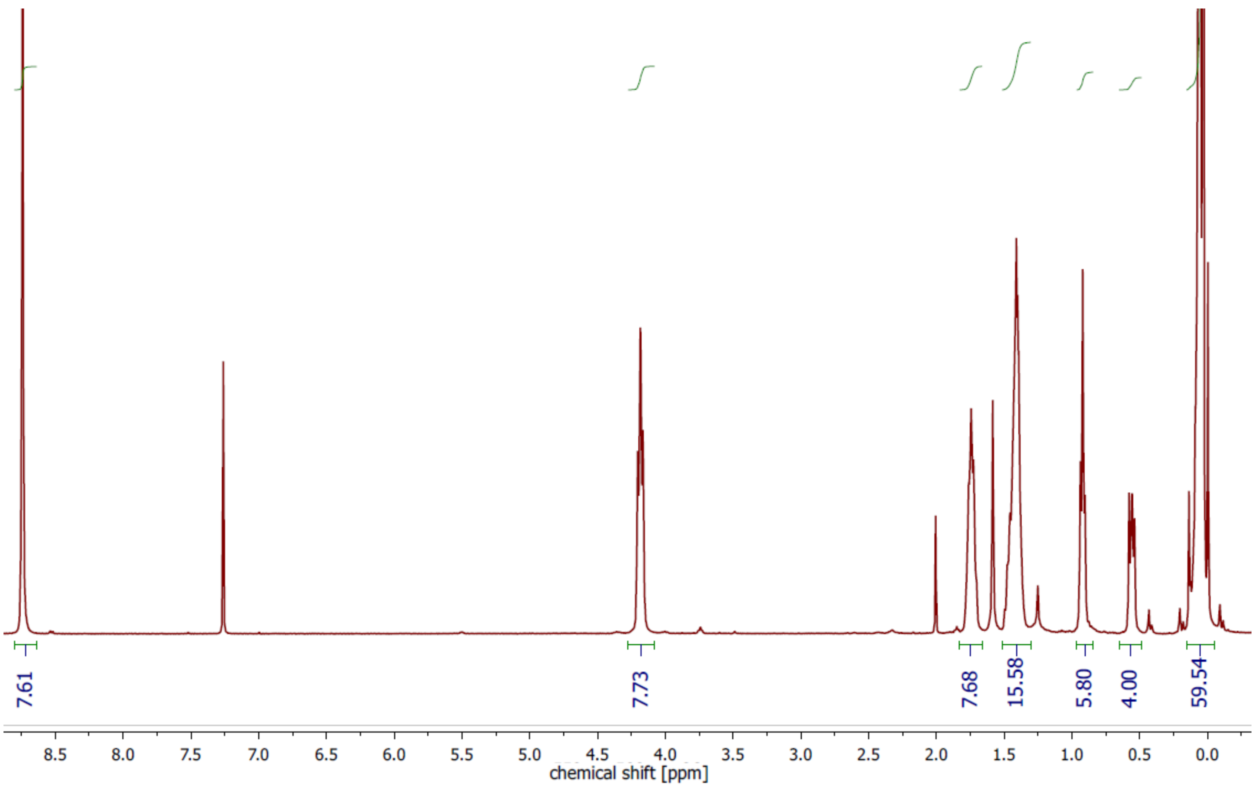

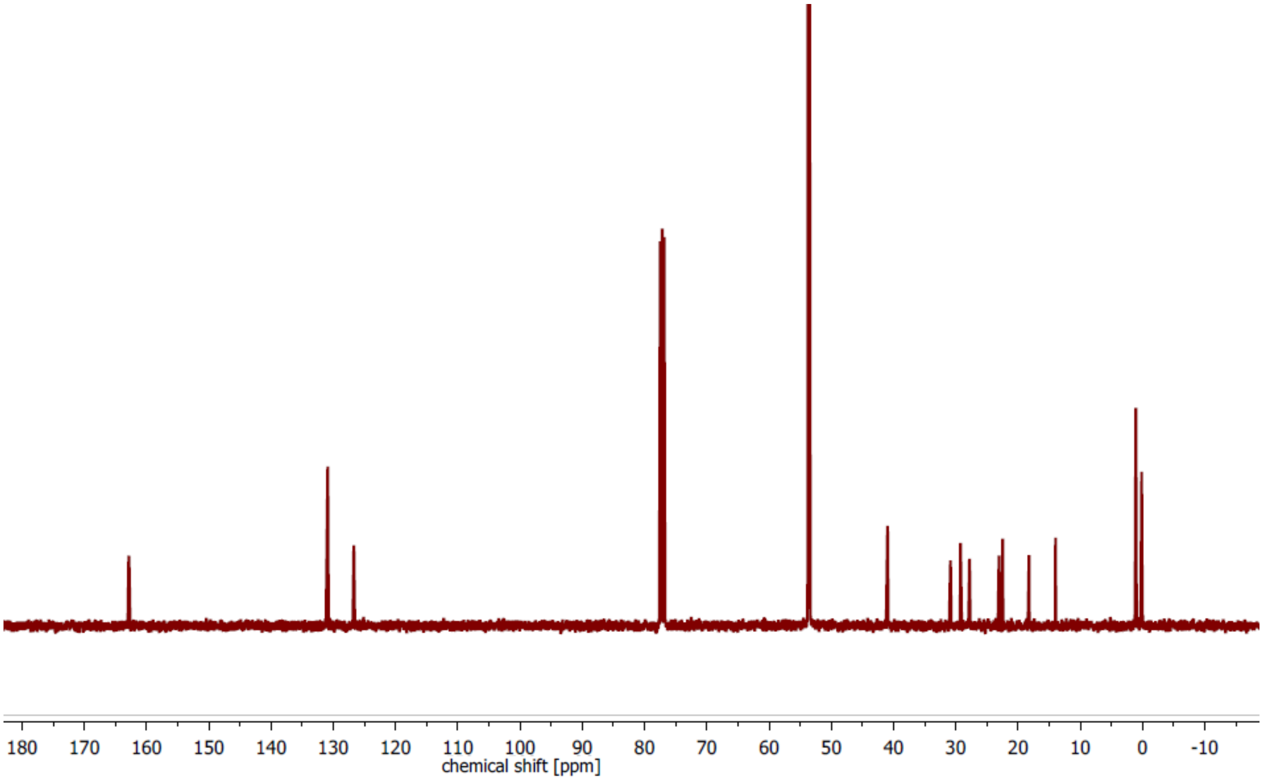

Figure S4. ^{1}H and ^{13}C NMR of **NDI-1** ($CDCl_3$)

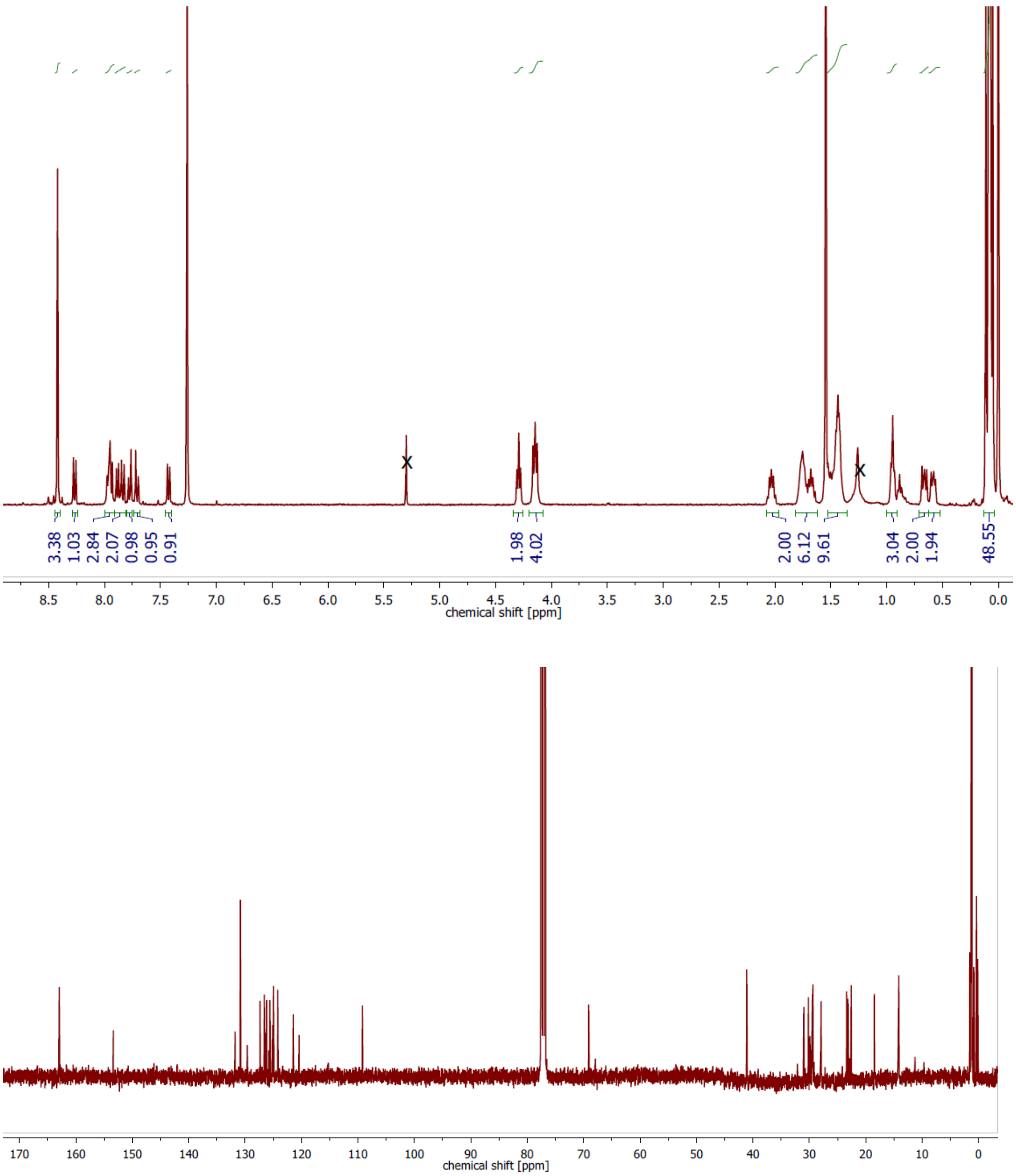

Figure S5. ^{1}H and ^{13}C NMR of **Pyr-Si$_8$-NDI** ($CDCl_3$)

4. Bulk co-assembly of homotelechelic NDI-1 with Pyr-*o*DMS, having a varying siloxane oligomer volume fraction

Mixing **Pyr-1** with **NDI-1** gives a mixture in which the two components have equal siloxane linker lengths (Si_8). To investigate the influence of the siloxane oligomer length on the bulk morphology and CT properties of the two-component material, mixtures of **NDI-1** with **Pyr-2** and **Pyr-3** were formed, denoted as **Pyr-2:NDI-1** and **Pyr-3:NDI-1**, respectively.

The thermal properties of the individual components (Figure S6) and mixtures were evaluated using DSC measurements (Figure S7A–C and Table 1). The DSC traces of the three mixtures are very comparable, showing a broad endothermic transition upon heating with a relatively low enthalpic energy (< 3.6 kJ mol^{-1}) compared to the melting temperatures of the individual components (Table 1), indicating an order-disorder transition (T_{ODT}) for all mixtures. Upon cooling, a single, sharp disorder-order transition temperature (T_{DOT}) is observed after which birefringent textures are formed, as detected by POM (Figure S7D–F). Liquid crystalline phases were observed for the **Pyr-2:NDI-1** and **Pyr-3:NDI-1** mixtures (Figure S7E–F), similar to the **Pyr-1:NDI-1** mixture.

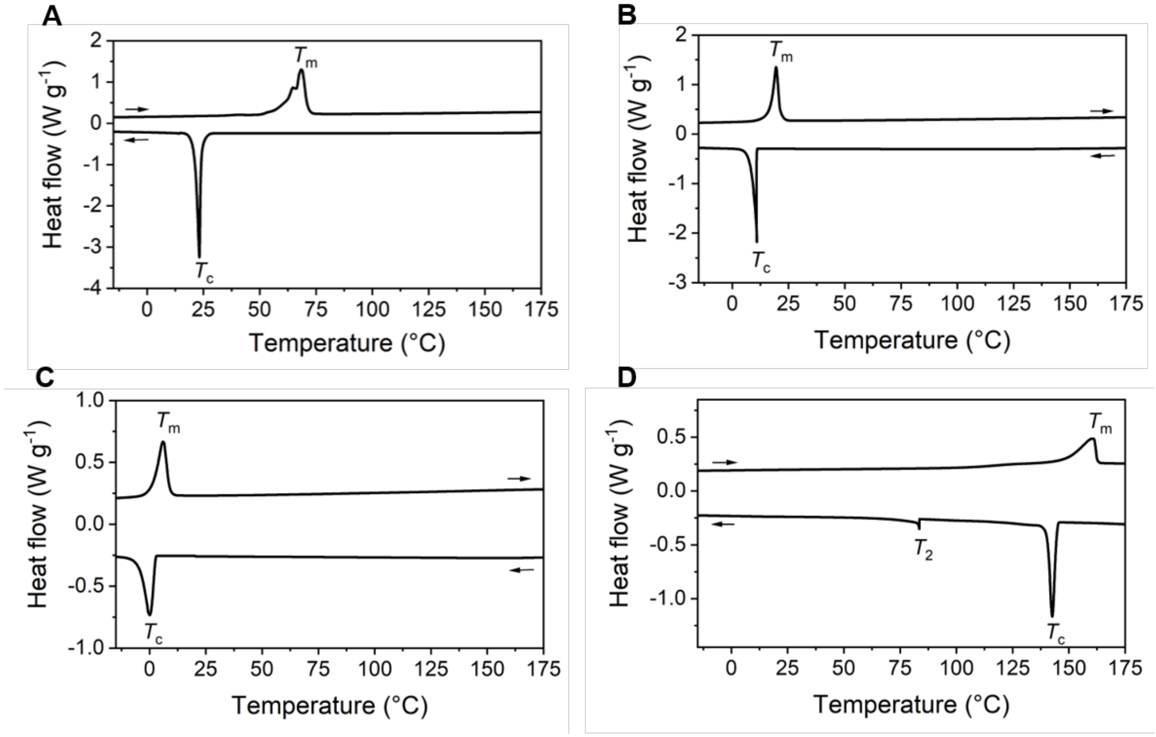

Figure S6. DSC traces (second heating and cooling cycle) of (A) **Pyr-1**, (B) **Pyr-2**, (C) **Pyr-3** and (D) **NDI-1**. A temperature ramp of 10 K min^{-1} was used. Endothermic heat flows have a positive value.

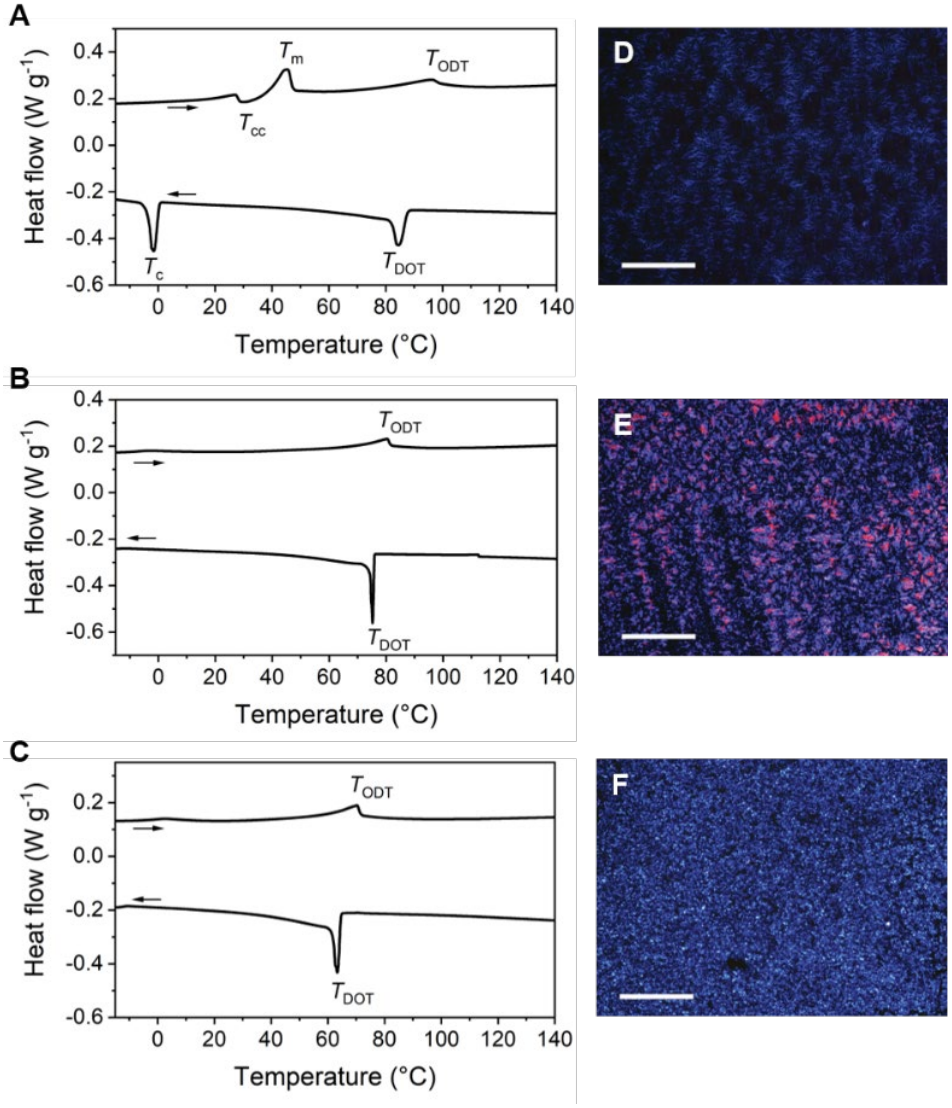

Figure S7. (A–C) DSC traces (second heating and cooling cycle) of (A) **Pyr-1:NDI-1**, (B) **Pyr-2:NDI-1** and (C) **Pyr-3:NDI-1**. A temperature ramp of 10 K min-1 was used. Endothermic heat flows have a positive value. (D–F) POM images (crossed polarizers) at room temperature of (D) **Pyr-1:NDI-1**, (E) **Pyr-2:NDI-1** and (F) **Pyr-3:NDI-1**. The material was placed in between two glass slides, heated to the isotropic state and cooled with 5 K min^{-1} to room temperature. Scale bar represents 250 μm.

We investigated the liquid crystalline order in the mixtures by medium- and wide-angle X-ray scattering (MAXS and WAXS) to determine the morphology and the extent of mixing (Figure S8). For the mixtures, co-assembly of the two oligomers can be concluded when a new, single nanostructured morphology is obtained. In contrast, a linear combination of the two components indicates a self-sorted, macrophase-segregated system.

Lamellar nanostructures were formed for all single components as follows from the scattering peaks at integer multiples of q^* in the transmission scattering profiles (Figure S8). This indicates the presence of a single lamellar structure for both **Pyr-2:NDI-1** and **Pyr-3:NDI-1** mixtures. Similar

to the **Pyr-1:NDI-1** mixture, the sharp scattering peaks in the high-*q* region of the transmission scattering profile of the single components disappear upon mixing and a single scattering peak at 18.3 nm^{-1} appears. Hence, the NDIs and pyrenes in the mixtures order by means of CT and π-stacking interactions in combination with microphase segregation. The presence of CT complexes in the nanostructure was confirmed by UV-Vis spectrometry (Figure S9).

Interestingly, the scattering peaks of the mixtures in the low-*q* region were much sharper than those of the individual **Pyr-1**, **Pyr-2** and **Pyr-3** block molecules. This suggests that a better-defined lamellar morphology is obtained for the mixtures compared to the Pyr-*o*DMS block molecules. Furthermore, the lamellar domain spacings of the mixtures were approximately the average of the domain spacing of the **NDI-1** and the pyrene block molecules (Table 1). A larger siloxane volume fraction of the Pyr-*o*DMS block molecule in the mixture gave rise to a larger domain spacing as the siloxane layer increases (Table 1, entries 5–7). Hence, we were able to tune the feature sizes of the CT material by changing the siloxane length in just one of the components in the mixture, while maintaining a high degree of order.

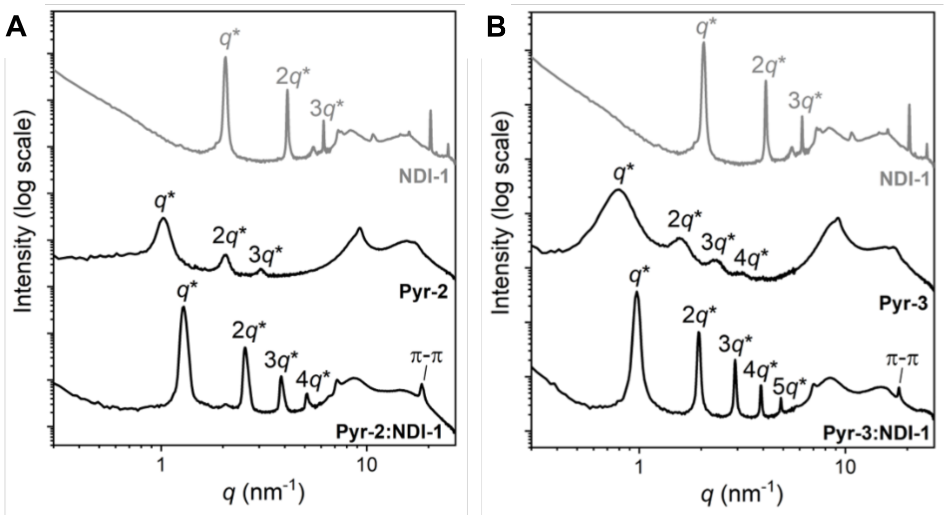

Figure S8. 1D transmission scattering profiles of (A) **Pyr-1:NDI-1** (bottom), **Pyr-1** (middle), **NDI-1** (top), (B) **Pyr-2:NDI-1** (bottom), **Pyr-2** (measured at 10 °C) (middle), **NDI-1** (top, grey) and (C) **Pyr-3:NDI-1** (bottom), **Pyr-3** (measured at −10 °C) (middle), **NDI-1** (top, grey). All block molecules were measured at room temperature if not stated otherwise.

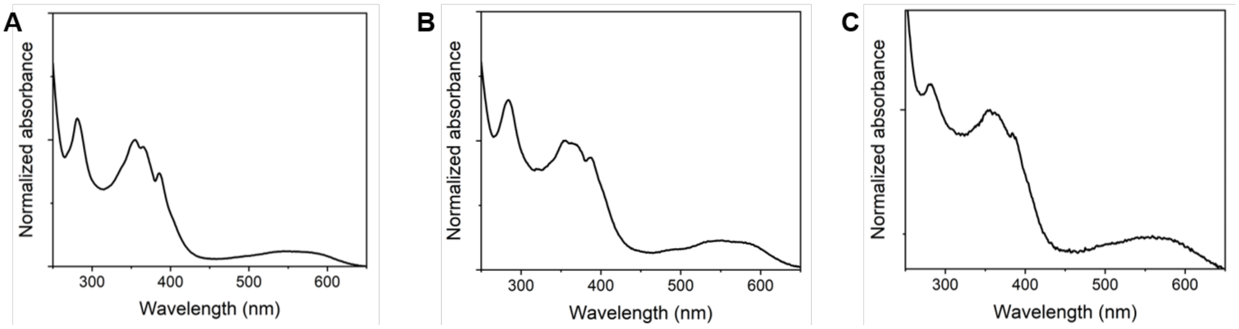

Figure S9. Solid state UV-vis spectra of (A) **Pyr-1:NDI-1**, (B) **Pyr-2:NDI-1** and (C) **Pyr-3:NDI-1**. Samples were prepared by spincoating (800 rpm) a 10 mg/mL solution of the mixture in chloroform onto the quartz substrate.

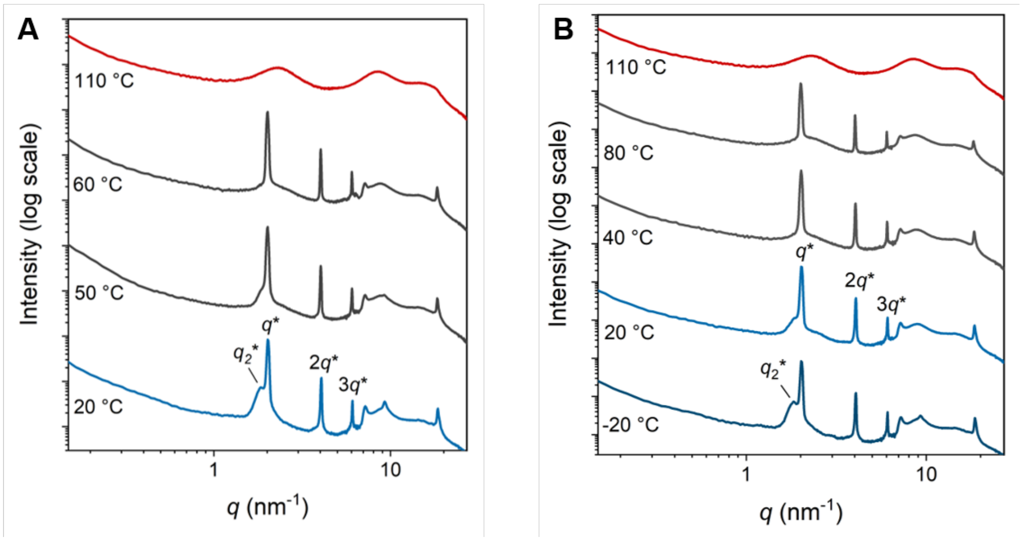

Figure S10. 1D transmission scattering profiles of **Pyr-1:NDI-1** upon (A) heating and (B) cooling with 5 K min^{-1}.

5. Influence of alkyl spacer on the morphology and CT properties of mixtures of Pyr-1 with NDI block molecules

The siloxane linker length of Pyr-*o*DMS in the mixtures had no effect on the order of the lamellar morphology and solely co-assembled structures were obtained, even with a difference of 32 siloxane repeating units between the NDI- and Pyr-*o*DMS block molecule. We also evaluated the effect of the carbon linker length on the co-assembly properties and morphology of the mixtures. For this, we mixed **Pyr-1**, having a C_5-alkyl linker, with reference molecules **NDI-2** and **NDI-3**, that have a C_{11}-alkyl linker, forming mixtures denoted as **Pyr-1:NDI-2** and **Pyr-1:NDI-3** (Figure S11A). The two mixtures both formed a macrophase segregated morphology, observed by a linear combination of the individual components in the 1D transmission scattering profiles (Figure S11). Thus, in order to obtain co-assembled nanostructures in the bulk, the alkyl linker lengths of the NDI- and Pyr-*o*DMS block molecules have to match to prevent self-sorting. These bulk assembly results are in accordance with results obtained in solution by Ghosh and coworkers, showing self-sorting of donor and acceptor chromophores due to an alkyl linker length mismatch.[5]

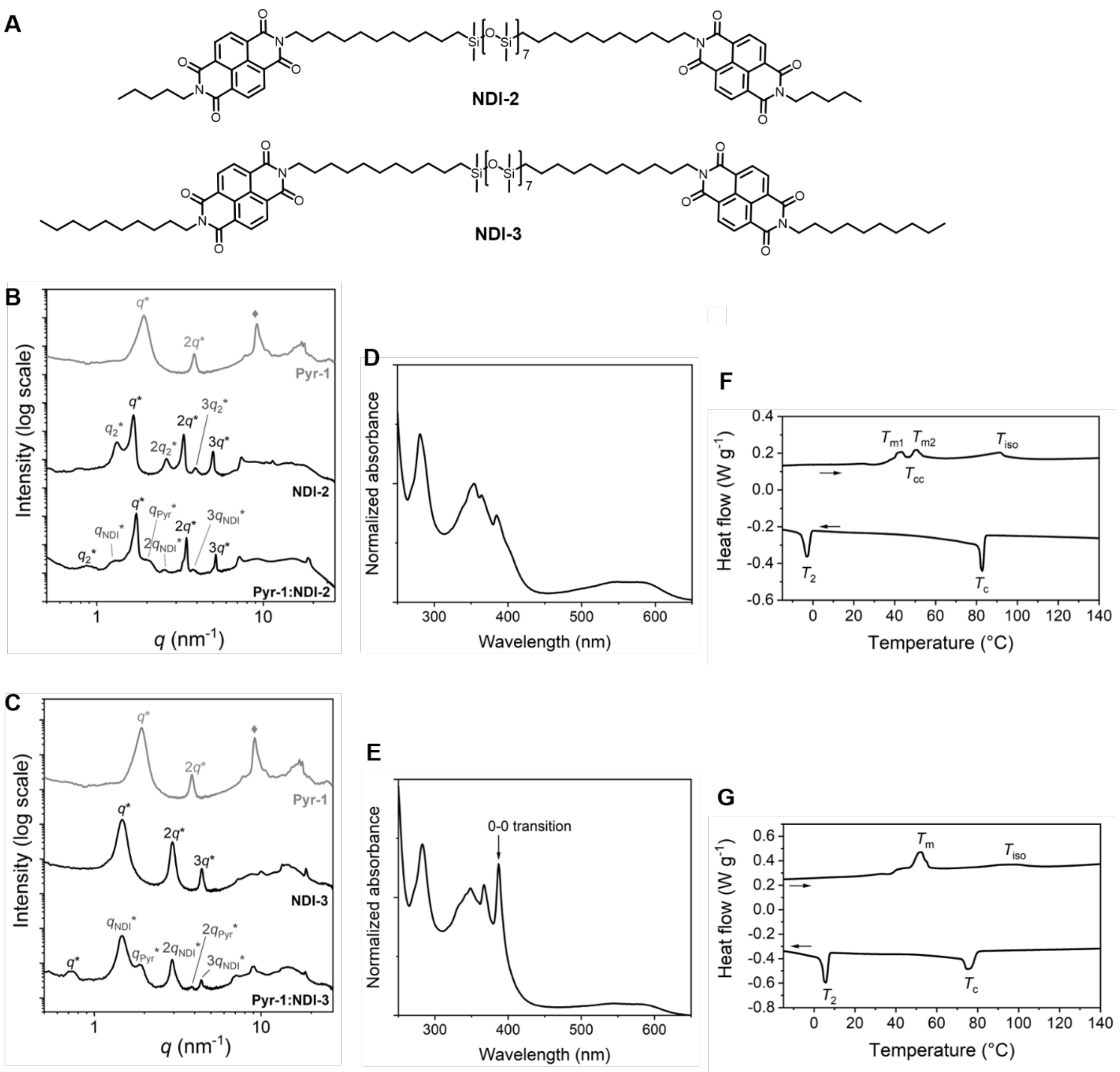

Figure S11. (A) Molecular structures of **NDI-2** and **NDI-3** reference block molecules. (B–C) 1D transmission scattering profiles of (B) **Pyr-1:NDI-2** (bottom), **NDI-2** (middle), **Pyr-1** (top, grey), (C) **Pyr-1:NDI-3** (bottom), **NDI-3** (middle), **Pyr-1** (top, grey). (D–E) Solid state UV-vis spectra of (D) **Pyr-1:NDI-2** and (E) **Pyr-1:NDI-3,** on a quartz substrate. 0-0 transition of the Pyr dimers is indicated. Samples were prepared by spincoating (800 rpm) a 10 mg/mL solution of the mixture in chloroform onto the quartz substrate which was annealed at 80 °C and cooled at 5 K min^{-1} to room temperature. (F–G) DSC traces (second heating and cooling run) of (F) **Pyr-1:NDI-2** and (G) **Pyr-1:NDI-3**. A temperature ramp of 10 K min^{-1} was used. Endothermic heat flows have a positive value.

6. Variable temperature X-ray measurements of Pyr-Si$_8$-NDI

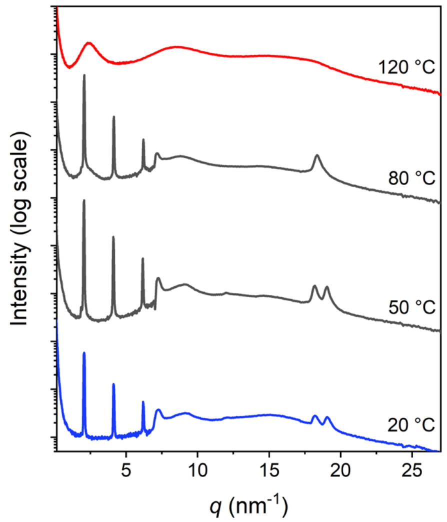

Figure S12. Variable temperature 1D transmission scattering profile of **Pyr-Si$_8$-NDI** upon cooling.

7. Changes in CT energy of Pyr-Si$_8$-NDI with pressure

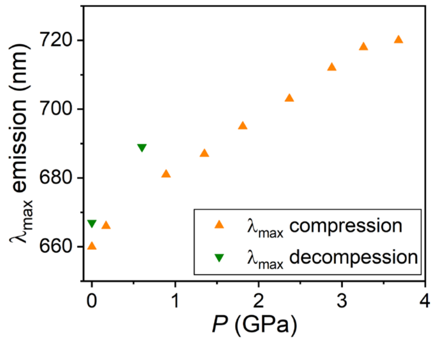

Figure S13. Peak maximum (λ_{max}) of the emission spectra as a function of pressure.

Linear correlation in Figure 4E:

To the set of experimental data consisting of the photon energy corresponding to the maximum in CT emission intensity at pressure P and the inverse stacking distance $1/d_{\pi 2}$ (P) also at pressure P , the following linear relation has been fitted using linear regression:

$$E_{CT}(P) = E_{CT}\left(d_{\pi_2} = \infty\right) + S\,\frac{1}{d_{\pi_2}(P)}$$

The fit is shown as the continuous red line in Figure 4E, with intercept $E_{CT}(d_{\pi 2} = \infty)$ = 4.4 eV and slope S = - 8 eV Å as optimized fit parameters.

8. UV-vis spectroscopy of Pyr-1:NDI-1 and Pyr-Si$_8$-NDI in solution

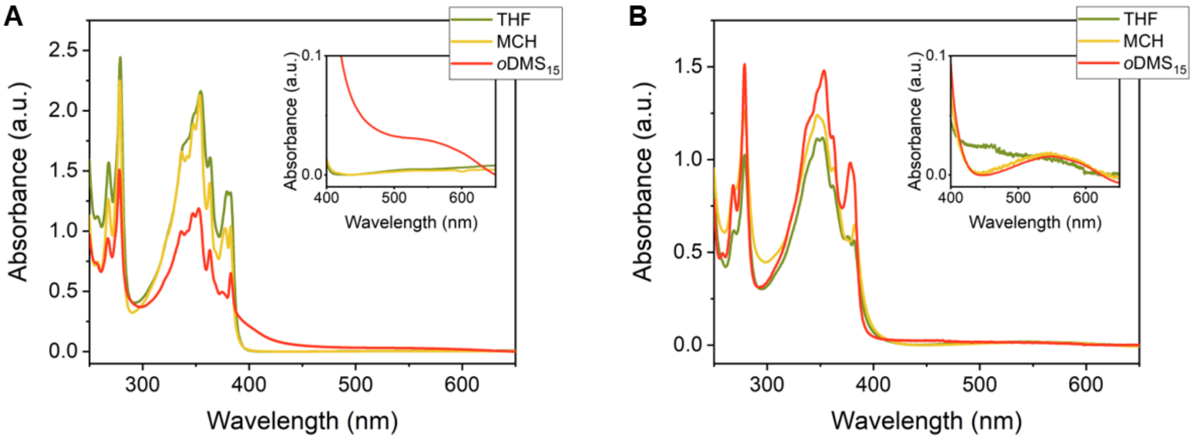

Figure S14. Absorption spectra of (A) **Pyr-1:NDI-1** and (B) **Pyr-Si$_8$-NDI** in THF (green), MCH (yellow) and siloxane (oDMS$_{15}$) solvent (red), 50 µM solutions. Insert shows the region at 400 to 650 nm showing a clear CT band for **Pyr-1:NDI-1** in oDMS$_{15}$ and **Pyr-Si$_8$-NDI** in MCH and *o*DMS$_{15}$.

9. References

1. B.A.G. Lamers, B.F.M. de Waal, E.W. Meijer, *J. Pol. Sci.*, 2021, 59, 1142–1150.
2. J. A. Berrocal, R. H. Zha, B. F. M. de Waal, J. A. M. Lugger, M. Lutz, E. W. Meijer, *ACS Nano,* 2017, **11**, 3733–3741.
3. G. J. Piermarini, S. Block, J. D. Barnett, R. A. Forman, *J. Appl. Phys.,* 1975, **46**, 2774.
4. Y. Seto, *Rev. High. Press Sci. Technol.*, 2010, **20**, 269–276.
5. M. R. Molla, A. Das, S. Ghosh, *Chem. - A Eur. J.* 2010, **16**, 10084–10093.
